# Triterpenoids and steroids isolated from Anatolian *Capparis ovata* and their activity on the expression of inflammatory cytokines

**DOI:** 10.1080/13880209.2020.1814356

**Published:** 2020-09-11

**Authors:** Isil Gazioglu, Sevcan Semen, Ozden Ozgun Acar, Ufuk Kolak, Alaattin Sen, Gulacti Topcu

**Affiliations:** a Department of Analytical Chemistry, Faculty of Pharmacy, Bezmialem Vakif University, Istanbul, Turkey; b Forensic Toxicology Laboratories, Institute of Forensic Sciences, Istanbul-Cerrahpasa University, Istanbul, Turkey; c Department of Biology, Faculty of Arts and Sciences, Pamukkale University, Denizli, Turkey; d Department of Analytical Chemistry, Faculty of Pharmacy, Istanbul University, Istanbul, Turkey; e Department of Molecular Biology and Genetics, Faculty of Life & Natural Sciences, Abdullah Gul University, Kayseri, Turkey; f Department of Pharmacognosy and Phytochemistry, Faculty of Pharmacy, Bezmialem Vakif University, Istanbul, Turkey

**Keywords:** Capparaceae, anti-inflammatory, anticholinesterase, fatty acid, secondary metabolites, olean-12-en-3β, 28-diol, 3β-pentacosanoate, structure elucidation, spectroscopy

## Abstract

**Context:**

*Capparis* L. (Capparaceae) is grown worldwide. Caper has been used in traditional medicine to treat various diseases including rheumatism, kidney, liver, stomach, as well as headache and toothache.

**Objective:**

To isolate and elucidate of the secondary metabolites of the *C. ovata* extracts which are responsible for their anti-inflammatory activities.

**Materials and methods:**

Buds, fruits, flowers, leaves and stems of *C. ovata* Desf. was dried, cut to pieces, then ground separately. From their dichloromethane/hexane (1:1) extracts, eight compounds were isolated and their structures were elucidated by NMR, mass spectroscopic techniques. The effects of compounds on the expression of inflammatory cytokines in SH-SY5Y cell lines were examined by qRT-PCR ranging from 4 to 96 µM. Cell viability was expressed as a percentage of the control, untreated cells.

**Results:**

This is a first report on isolation of triterpenoids and steroids from *C. ovata* with anti-inflammatory activity. One new triterpenoid ester olean-12-en-3β,28-diol, 3β-pentacosanoate (**1**) and two new natural steroids 5α,6α-epoxycholestan-3β-ol (**5**) and 5β,6β-epoxycholestan-3β-ol (**6**) were elucidated besides known compounds; oleanolic acid (**2**), ursolic acid (**3**), β-sitosterol (**4**), stigmast-5,22-dien-3β-myristate (**7**) and bismethyl-octylphthalate (**8**). mRNA expression levels as EC_10_ of all the tested seven genes were decreased, particularly CXCL9 (19.36-fold), CXCL10 (8.14-fold), and TNF (18.69) by the treatment of 26 µM of compound **1** on SH-SY5Y cells.

**Discussion and conclusions:**

Triterpenoids and steroids isolated from *C. ovata* were found to be moderate-strong anti-inflammatory compounds. Particularly, compounds **1** and **3** were found to be promising therapeutic agents in the treatment of inflammatory and autoimmune diseases.

## Introduction

*Capparis* L. (Capparaceae) (caper) is represented by 200–250 species which have been widely grown in the world including the subtropical climate zones, particularly throughout Mediterranean basin. They have been cultivated and used for nutritional, cosmetic and pharmaceutical purposes as well as for animal feeding, to prevent soil erosion and landscaping. Since antiquity, caper buds, fruits, bark, roots and seeds have been used in traditional medicine to treat various diseases including rheumatism, kidney, liver, stomach, as well as headache and toothache (Aichi-Yousfi et al. 2016, Tlili et al. 2009, 2011). Scientific investigations have shown that capers have various biological activities, such as antioxidant, antimicrobial, anti-inflammatory, immunostimulant, antidiabetic, antitumor, and antisclerosis (Panico et al. 2005; Argentieri et al. 2012).

In Turkey, the genus *Capparis* L. is widely grown and distributed namely by two species; *C. ovata* Desf. and *C. spinosa* L. (Coode 1965). They have been known as gabara, kebere, kapari, gebre, gebele, çaltıdikeni with folkloric uses, such as diuretic, constipation and tonic as well as culinary purposes (Baytop 1984), and they are commonly consumed in Turkey, especially in pickled forms (called ‘turşu’ in Turkey) in Aegean-Mediterranean and throughout centre parts of Anatolia. Immature flower buds are part of human nutrition; they are used in sauces and pizzas and are served with fish, meat and salads due to its aroma similar to mustard and pepper. Since the last two decades, *Capparis* species grown in Turkey have been phytochemically investigated by only a limited number of authors (Calis et al. 1999, 2002; Matthaus and Ozcan 2005; Duman and Ozcan 2014). Anatolian *C. spinosa* has been studied for its secondary metabolites by Calis group (1999, 2002) which afforded new 1H-indole-3-acetonitrile glycosides and (6S)-hydroxy-3-oxo-α-ionol glucosides (Calis et al. 2002). *Capparis* species were further investigated for pharmacological/biological activities (Arslan et al. 2010; Bektas et al. 2012; Ozgun-Acar et al. 2016).

In another study, *C. ovata* seeds were found to be rich in protein, oil, and fibre (Akgul and Ozcan 1999). Also, many fatty acids in the seeds including oleic, palmitic, linoleic, lauric, gadoleic, myristic, arachidic, capric, behenic, and lignoceric acids were determined at important percentages. The protein amount of *C. ovata* seeds in Turkey was determined to be 22% (Tlili et al. 2009) as well as mineral ingredients of the shoots, flowers, buds, fruits, and seeds were found to be high especially for Pb, K, P, Ca, Mg, Zn, and Na (Ozcan 2005).

Essential oil composition and antioxidant activities of buds and leaves of *C. ovata* var. canescens, cultivated in Turkey, were reported (El-Ghorab et al. 2007). Results, obtained by Arslan et al. (2010), concluded that the methanol extract of *C. ovata* flower buds was found to be a potentially antinociceptive agent which acts as both at the peripheral and central levels in mice. In the same study, the fruit methanol extract also showed antinociceptive activity. An *in vivo* study reported that injections of *C. ovata* extracts modulate brain oxidative toxicity and epileptic seizures in pentyl tetrazole-induced epileptic rats (Naziroglu et al. 2013).

Some phytochemical characteristics such as total phenolic content, vitamin C, total carotenoid content and antioxidant activity of 44 wild grown caper (*C. ovata*) genotypes sampled in Coruh valley located in the Northeastern part of Turkey were determined (Dogan et al. 2014).

Our group (Sen et al. 2014) has previously reported the anti-neuroinflammatory effect of butanol subextract of *C. ovata* obtained from fermented water extract which was used as an alternative and complementary treatment for multiple sclerosis (MS) in Anatolia. Therefore, our studies focussed on a search for antineuroinflammatory effects by treating C57BL6 mice with the prepared *C. ovata* extracts which suppressed inflammatory cytokine expression and ameliorated experimental autoimmune encephalomyelitis design of multiple sclerosis (Ozgun-Acar et al. 2016). In a continuing study, a potential therapeutic activity of a natural plant steroid; stigmast-5,22-dien-3β-ol myristate, obtained from *C. ovata*, was tested and found to be a fairly promising agent for the treatment of MS (Ozgun-Acar et al. 2017).

The present study isolated and elucidated the structure of the secondary metabolites from *C. ovata* extracts, prepared from different parts (organs) of the plant, which are found to be responsible for their anti-inflammatory and anticholinesterase activities.

## Materials and methods

### Chemicals and reagents

For the cholinesterase inhibitory test assays, DTNB (5,5-dithiobis-[2-nitrobenzoic acid]) (99.9%) and standard anticholinesterase compound galanthamine hydrobromide (>99%) were purchased from Sigma-Aldrich (Steinheim, Germany). The electric eel acetylcholinesterase (AChE, Type-VI-S, EC 3.1.1.7, 425.84 U/mg) and horse serum butyrylcholinesterase (BuChE, EC 3.1.1.8, 11.4 U/mg) were purchased from Sigma (Steinheim, Germany). The substrate of AChE, acetylthiocholine iodide (>99%) from Applichem (Darmstadt, Germany), and the substrate of BuChE, butyrylthiocholine iodide (>99%) from Fluka (Steinheim, Germany) were purchased.

The chemicals and solvents, sodium carbonate, sodium hydrogen phosphate, sodium dihydrogen phosphate, and hexane, dichloromethane, ethylacetate, ethanol, methanol, butanol, and silica gel for column chromatography (1.07734) and silica gel 60 F254 TLC plates (1.05554), potassium hydroxide, sodium sulphate, hydrochloride acid, and diethyl ether were purchased from Merck (Darmstadt, Germany).

### General experimental procedures

The UV (Shimadzu UV-1601), NMR (600 MHz for ^1^H-NMR, 150 MHz for ^13^C-NMR, Varian VNMRS), GC-MS/MS (Thermo Scientific TSQ, Thermo TR-BD MS) were used for the analyses. A pH metre (Thermo), an ultrasonic bath (Elma S15), a vortex (LMS Co., Ltd.), and a BioTek Power Wave XS (Winooski, VT, USA) were used for the activity assays.

### Plant material

*C. ovata* was collected at the beginning of June 2012 in Burdur (Southern Turkey). A voucher specimen was identified by Dr. Mehmet Çiçek and was deposited in the Herbarium of the Faculty of Arts and Sciences at Pamukkale University (PAMUH2012000006300), Denizli, Turkey.

### Preparation of the extracts

Each part of the collected plant was separated, dried at room temperature; fresh flowering buds of *C. ovata* (1.795 kg), fruits (1.280 kg), flowers (221.9 g), leaves (418.7 g), and stems (934.8 g) were cut to pieces and grounded in a blender. They were extracted directly with hexane/dichloromethane (1:1) five times (each for 24 h). After filtration, the solvents were evaporated to dryness under vacuum to afford the separate five extracts.

### Isolation of the secondary metabolites

Totally five extracts were obtained, from different parts of the plant by exhausting with hexane-dichloromethane (1:1), their amounts and yields as follows: the buds (CHDB) (12.92 g, 0.72% yielded), fruits (CHDFr) (20 g, 1.56% yielded), flowers (CHDFl) (2.17 g, 0.97%, yielded), leaves (CHDL) (1.25 g, 0.5% yielded), and stems (CHDS) (1 g, 0.10% yielded). Except for the leaves extract, due to low yield, the other four extracts were subjected to a silica gel column separately to fractionate and isolate pure compounds. Elution was started with petroleum ether (40–60°), a gradient of dichloromethane was added with 10% increments into reaching 100% dichloromethane, followed by acetone increments with 10% up to 100% acetone, and final elutions are completed by 5%, 10%, 50% and 100% methanol additions. Similar fractions were combined and evaporated. When necessary, obtained compounds were purified through Sephadex LH-20 columns and/or prep. TLC (preparative thin layer chromatography) plates.

The buds of *C. ovata* hexane-dichloromethane (1:1) (CHDB) extract afforded six compounds (**1–6**) which were obtained from its different fractions; the new compound was obtained from fractions (23–27) and purified by preparative TLC using petroleum ether:dichloromethane (4:6) and its structure was elucidated as olean-12-en-3β,28-diol, 3β-pentacosanoate (= 3β-pentacosanoylolean-12-en-28-ol) (**1**) (24 mg). Subsequently, from the three fractions (11), (17) and (28-29), β-sitosterol (**4**) was isolated and purified (22.7 mg) by prep TLC using a dichloromethane:acetone (9.5:0.5) solvent mixture. From fractions (35–40): oleanolic acid (**2**) (12 mg) and ursolic acid (**3**) (8 mg) which were purified by preparative TLC using dichloromethane:acetone (9:1), while from fractions (14-15), two steroids 5α,6α-epoxycholestan-3β-ol (**5**) and 5β,6β-epoxycholestan-3β-ol (**6**) (total 25 mg) were purified by prep. TLC using petroleum ether:dichloromethane (9:1) solvent systems.

From the fruit extract (CHDFr), two compounds were obtained; one was the steroid ester stigmast-5,22-dien-3β-myristate (**7**) (21 mg), and a fatty acid phythalate bismethyl-octylphthalate (**8**) (5 mg) through isolating from fraction 4 [by prep. TLC with petroleum ether:dichloromethane (3:2), and fraction 13 (hexane:dichloromethane, 7:3)], respectively.

### Esterification of total fatty acids

For the derivatization of free fatty acids: The seed oil (30 mg) was dissolved in 1 mL of toluene and H_2_SO_4_ in 2 mL of methanol was then added. The mixture was left for 24 h at 50 °C, then 5%, 5 mL of NaCl solution was added and the required esters were extracted with hexane (2 × 5 mL), then the organic layer was separated, and the hexane layers were washed with 2%, 4 mL of potassium bicarbonate solution, dried over anhydrous Na_2_SO_4_ and filtered. The organic solvent was removed on a rotary evaporator to obtain fatty acid methyl esters.

### Gas chromatography-mass spectrometry (GC/MS)

Thermo Scientific TSQ system and a Thermo TR-BD MS (30 m × 0.25 mm ID × 0.25 µm) were used for the GC-MS/MS analyses. The carrier gas was helium at a flow rate of 1.0 mL/min. The oven temperature was held at 50 °C for 5 min, then increased up to 250 °C with 5 °C/min increments and held at this temperature for 10 min. Ion source temperature was 230 °C. The injection volume was 1 μL with a split ratio of 1:20. Mass range was from *m/z* 50–650 amu. Scan time was 0.5 s with 0.1 s interscan delays. Identification of components was based on GC retention indices and computer matching with the NIST Library.

### Determination of anticholinesterase activity

Acetylcholinesterase (AChE) and butyrylcholinesterase (BuChE) inhibitory activities were established by slightly modifying spectrophotometric method developed by Ellman et al. (1961), only for the extracts.

### Cell culture

The human neuroblastoma cell line SH-SY5Y was obtained from the American Type Culture Collection (ATCC, USA). The cells were cultured in DMEM-F12 supplemented with 10% FBS and 1% penicillin/streptomycin mixture in a humidified atmosphere of 95% air with 5% CO_2_ at 37 °C and were subcultured twice a week.

### Cytotoxicity assay

SH-SY5Y cells were seeded in 96-well plates at a density of 1 × 10^3^ cells/mL culture medium. (DMEM-F12) as mentioned in the cell culture part. After 24 h incubation, the cells were treated with varying concentrations (ranging from 4 to 96 µM) of the purified secondary metabolites (PSM). An equal amount of medium without PSM was added to untreated cells (control). PSM-treated and control cells were incubated for 24 h at 37 °C in humidified 5% CO_2_ atmosphere. Following incubation, the medium was replaced by 0.5% crystal violet solution (w/v; in 50% ethanol). The dye absorbed by live cells was extracted with sodium citrate (0.1 M in 50% ethanol). Absorbance was read at 630 nm. Cell viability was expressed as a percentage of the control, untreated cells (Ozgun-Acar et al. 2017).

### RNA isolation and cDNA synthesis

Total RNA was extracted from SH-SY5Y cells by using RNeasy Plus mini kit (Qiagen) according to the manufacturer’s instruction with slight modifications. Elution was performed with 40 µL RNase-free water. After elution, the RNA concentration was determined using a Nanodrop (MaestroNano micro-volume Spectrophotometer, USA), and the RNA was reverse transcribed using Easy Script cDNA Synthesis Kit (ABM). The reaction mixture was incubated for 50 min at 50 °C followed by termination by heating at 5 min 85 °C. cDNA was stored at −80 °C for further use (Ozgun-Acar et al. 2017).

### Quantitative RT-PCR

Quantitative Real Time PCR (qRT-PCR) analysis was performed using SYBR Green qPCR Master Mix (GM, Taiwan) in an Exicycler 96 Real Time Quantitative Thermal Block PCR System (Bioneer, Daejeon, Korea) for each gene. The mRNA levels of genes (CCL5, CXCL9, CXCL10, HIF1A, IL6, NF-κB1, TNFα) were determined by qRT-PCR. Beta-actin was chosen from the group of housekeeping genes as the least varying reference gene. The qPCR using custom designed primers for the genes listed in Supplementary Table S1 (Ozgun-Acar et al. 2016).

### Statistical analysis

Statistical analysis was performed using the Minitab 13 statistical software package (Minitab Inc. State College, PA, USA). All results were expressed as means including their Standard Error of Means (SEM). Comparison between groups was performed using Student’s *t*-test, and *p* < 0.05 was selected as the level required for statistical significance.

## Results and discussion

### Determination of fatty acids by using GC/MS

It was significant that the all investigated extracts, prepared from different parts of *C. ovata* were found to be rich in sterols and terpenoids as well as long chain fatty acids Thus, fatty acid composition of the seeds of mature fruits (CHDFr) extract was analysed by GC-MS, and linoleic acid was found to be the major fatty acid (30.90%) as an omega-6 fatty acid, which is one of the essential fatty acids beside another omega-6 acid; arachidonic acid with a very low percentage. Other major acids were 2-methyl-2-pentenoic acid (19.10%), oleic acid (14.60%) and its trans isomer elaidic acid (*t*-Δ^9^-octadecenoic acid = *t*-oleic acid) (14.40%) while the relative abundance of palmitic acid was found to be 7.50% (Table 1). As an omega-3 fatty acid, only α-linolenic acid was present in this fatty acid composition, but with a fairly low percentage (1.03%). The fatty acid contents of the seeds of *C. ovata* and *C. spinosa* collected 11 different localities in Turkey have been previously investigated by Matthias and Ozcan (2005) in detail. In comparison to the fatty acid composition of those seeds with that of fruits extract of the *C. ovata* (CHDFr extract), some similarities were found (Table 1), especially for linoleic and oleic acid percentages.

**Table 1.** Fatty acid composition of mature fruit (CHDFr) extract.

R_T_^a^	Constituent^b^	Composition (%)^c^	Formula
16.61	2-Methyl-2-pentenoic acid	19.10	C_6_H_10_O_2_
17.13	2,2-Dimethyl pentanoic acid	7.30	C_7_H_14_O_2_
23.14	Lauric acid (12:0)	0.03	C_12_H_24_O_2_
27.43	Myristic acid (14:0)	0.33	C_14_H_28_O_2_
31.34	Palmitic acid (16:0)	7.50	C_16_H_32_O_2_
31.78	Palmitoleic acid (16:1, Δ^9^)	1.90	C_16_H_30_O_2_
32.80	9,12-Hexadecadienoic acid (16:2, Δ^9,12^)	0.13	C_16_H_28_O_2_
33.18	Margaric acid (17:0)	0.03	C_17_H_34_O_2_
34.96	Stearic acid (18:0)	1.90	C_18_H_36_O_2_
35.26	Oleic acid (18:1, Δ^9^)	14.60	C_18_H_34_O_2_
35.38	Elaidic acid (18:1, Δ^9^)	14.40	C_18_H_34_O_2_
36.02	Linoleic acid (18:2, Δ^9,12^) (ω-6)	30.90	C_18_H_32_O_2_
37.09	α-Linolenic acid (18:3, Δ^9,12,15^) (ω-3)	1.03	C_18_H_30_O_2_
38.32	Arachidonic acid (20:4, Δ^9,12,15^) (ω-6)	0.40	C_20_H_32_O_2_
38.60	11-Eicosenoic acid (20:1, Δ^5,8,11,14^) (ω-6)	0.16	C_20_H_38_O_2_
41.45	Behenic acid (21:0)	0.13	C_22_H_44_O_2_

^a^Retention time (in minutes). ^b^Compounds listed in order of elution from Thermo TR-BD MS (30 m × 0.25 mm ID × 0.25 µm). ^c^Percentage of relative weight.

### Isolation of the secondary metabolites

**Figure 1.**
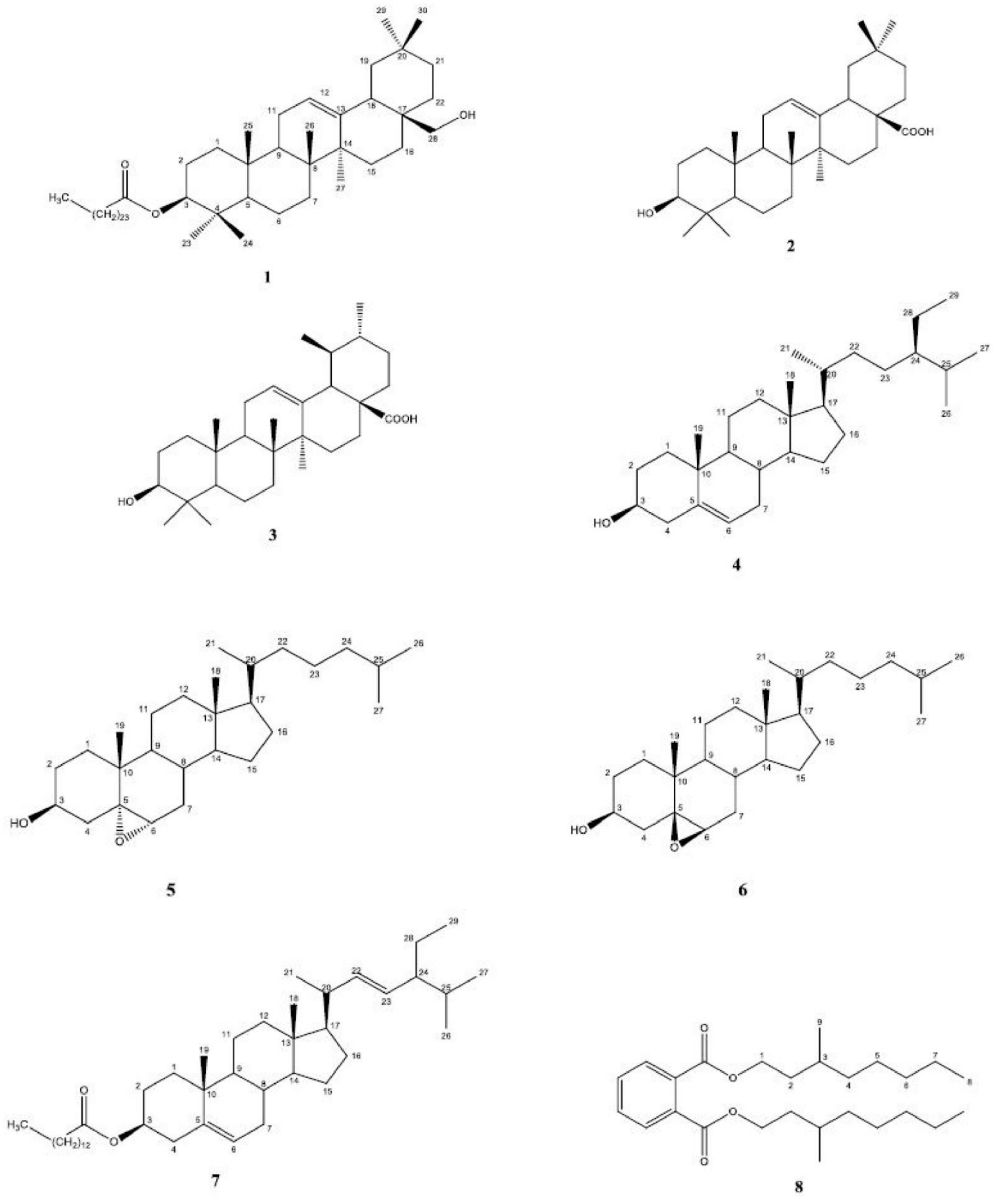
Isolated compounds from* Capparis ovata.*

Except the leaves extract, each extract was applied to Si-gel column chromatography, separately, and eluted with non-polar to polar solvent systems to fractionate. Similar fractions were merged after TLC control and then applied to shorter Si-gel columns and preparative TLC to yield pure compounds. In addition to a new triterpenoid ester (**1**), two known triterpenoids oleanolic acid (**2**), and ursolic acid (**3**) (Kolak et al. 2009), the three steroids (**4–6**) (Carvalho et al. 2009; Sen et al. 2017) were isolated from the buds of *C. ovata* hexane-dichloromethane (1:1) extract, while the steroid myristate ester (**7**) (Sen et al. 2017) was obtained from the fruits of hexane-dichloromethane (1:1) extract beside a fatty acid bis (7-methyloctyl) phthalate (**8**) (see Figure 1) (Bindu et al. 2017).

The compound (**1**) was isolated for the first time from *C. ovata* and identified as a new compound by our group. The ^13^C NMR spectrum (Table 2) revealed an oleanane triterpene skeleton linked to a long chain ester moiety (30 carbon belonging to the main triterpene skeleton and 25 carbon attributed to a long chain) which was verified by the observation of 7 methyl singlets, and an end methyl triplet of the long chain ester moiety in the ^1^H NMR spectrum (Table 2). Instead of a hydroxyl group at C-3, the presence of an ester moiety was followed by the appearance of a signal at δ 4.50 (1H, dd, *J* = 5.5 and 10.2 Hz, H-3) along with a characteristic double bond signal between C-12 and C-13 at δ 5.19 (1H, t, *J* = 2.5 Hz, H-12). In addition, there were two doublets as an AB pair of two proton signals attributed to a primary hydroxyl group, preferably located either at C-28 or one of the C-4 methyls. As it is known, other methyl groups in an oleanane structure also have the possibility to carry this hydroxyl group, but with a less possibility than C-28 methyl. The primary hydroxyl protons were observed at δ 3.55 (1H, d, *J* = 10.5 Hz, H-28a) and 3.22 (1H, d, *J* = 10.5 Hz, H-28b), and their attached carbon appeared at δ C 69.7 was followed by an HMQC (hetero multiple quantum correlation) experiment (Table 2). The location of this carbon to be C-28 was followed by an HMBC (hetero multiple bond correlations) experiment, exhibiting a three-bond away correlations with H-18 (1.90 dd), and H-22 (1.59, m) (Table 2), the loss of CH_2_OH [M-31]^+^ peak from the molecular ion peak (M)^+^ at *m/z *806 was observed at *m/z* 413 [M^+^ – pentacosanoyl (C_25_H_49_O) – (CH_2_OH)]^+^ The presence of a long chain fatty acid ester in the structure was also verified by the observation of the fragment ion at *m/z* 391 [(M-1)^+^ -erythrodiol- CH_2_OH]^+^ originating from the molecular ion corresponding to a molecular formula C_55_H_98_O_3_ in the HR-MS spectrum. Based on all the spectral data, structure of the compound (**1**) was elucidated to be olean-12-en-3β,28-diol, 3β-pentacosanoate (3β-pentacosanoylolean-12-en-28-ol) which was obtained for the first time from nature. It should be named as erythrodiol 3β-pentacosanoate (IUPAC name: 3*S*,4a*R*,6a*R*,6b*S*,8a*S*,12a*S*,14a*R*,14b*R*)-8a-(hydroxymethyl)-4,4,6a,6b,11,11,14b-heptamethyl −1,2,3,4a,5,6,7,8,9,10,12,12a,14,14a-tetradecahydropicen-3-ol, pentacosanoic acid).

**Table 2.** ^1^H-NMR, ^13^C-NMR, HMQC, HMBC spectroscopy data acquired to compound **1**.

Position C	δ_H_ (ppm)	δ_C_ (ppm)	HMQC (C→H)	HMBC (C→H)
1	1.62 m	38.1	H-1	H-2
2	1.63 m	23.4	H-2	H-1
3	4.50, dd (*J=*5.5;10.2)	81.0	H-3	H-2
4	–	37.6	–	H-2′
5		55.1	H-5	H-7
6		18.1	H-6	H-4, H-7
7	1.54, m	32.4	H-7	H-5
8	–	39.8	–	H-6
9		49.0	–	H-26
10	–	36.8	–	H-5, H-6
11	1.88 dd (*J=*2.5;11 Hz)	23.4	H-11	H-9, H-7
12	5.19 t (*J* = 2.5 Hz)	122.1	H-12	H-11, H-18
13	–	144.1	–	H-11, H-12
14	–	41.6	–	H-26
15	1.02	25.1	H-15	H-27, H-28
16	1.10	25.2	H-16	H-28
17	–	31.8	–	H-21
18	1.90	42.2	H-18	H-19
19	1.55	46.3	H-19	H-18
20	–	34.0	–	H-18
21	1.59	30.9	H-21	H-22
22	1.61	38.1	H-22	H-19
23	1.17, s	27.9	H-23	H-24
24	0.88, s	16.6	H-24	H-1
25	0.87, s	15.4	H-25	H-5, H-3
26	0.94, s	16.6	H-26	H-14, H-9
27	0.96, s	25.8	H-27	
28a	3.55, d, *J=*10.5 Hz	69.7	H-28	H-18
28b	3.20, d, *J=*10.5 Hz			
29	1.25, s	33.1	H-29	H-30
30	0.89, s	23.5	H-30	–
-COO-	–	173.6	–	H-2, H-2′, (CH_2_)n
CH_3_	0.85, t (*J=*7.5 Hz)	14.0	H-25′	–
-CH_2_-	2.3, t (*J=*7.5 Hz)	34.5	H-2′	(CH_2_)n
(CH_2_)n	1.50–1.65, m	29.7	H3′ – H24′	–

Compounds (**2**) and (**3**) are very well known triterpenoids (oleanolic acid and ursolic acid), and β-sitosterol (**4**) is a very common steroid in the plant world.

The two steroids 5α,6α-epoxycholestan-3β-ol (**5**) and 5β,6β-epoxycholestan-3β-ol (**6**) were isolated from CHDB extract. Their structure elucidation was made based on NMR spectral data. Literature screening showed us the two steroids were not isolated before from nature. However, there is a publication which shows their syntheses carried out by Carvalho et al. (2009). The ^13^C NMR and ^1^H NMR spectra (in CDCl_3_) of both isomers indicated their cholestane skeleton with 27 C atoms and characteristic 5 methyl signals. In fact, an APT experiment (^13^C NMR) clearly exhibited the presence of 5 methyl, 11 methylene, 8 methine and 3 quaternary carbon signals for each stereoisomer resonating 11.80–69.41 ppm. In their ^1^H NMR spectra (600 MHz, CDCl_3_), two methyl signals are very significant for CH_3_-18 and CH_3_-19 protons appeared as singlets, respectively at δ 0.61 and 1.06 for steroids. The other three methyl signals were observed as doublets at δ 0.89 (CH_3_-21), 0.85 (CH_3_-26) and δ 0.83 (CH_3_-27) with *J* values of about 6.6 Hz for compound **5**. In the ^13^C NMR spectrum (150 MHz, CDCl_3_) (APT experiment) the five methyl carbons were observed at δ 11.95, 15.90, 18.69, 19.00, 19.80 and three oxygenated signals appeared at δ 68.71, 65.68, and 59.28. The first signal attributed to the presence of a methine carbon signal which bears a secondary hydroxyl group, possibly at C-3. Because, a substituent, particularly a hydroxyl or ester group in the natural steroids and triterpenoids first prefers to be located at C-3, biogenetically. ^1^H NMR chemical shift of H-3α (3.91, ttt, *J* = 11.4, 11.5, 5.4, 5.3) with significant *J* coupling values of the vicinal carbon protons (C-2 and C-4), verified the location of the secondary hydroxyl group at C-3 at β position. The other two oxygenated carbon signals appeared as a quaternary C at δ 65.68, and δ 59.28 as a methine C respectively, which were assigned to the presence of an epoxy group between C-5 and C-6, and H-6 signal resonated at 2.91 ppm as a doublet (d, *J* = 6.1 Hz) indicating its beta location, therefore, epoxy bonds should be α. In the APT experiment, six methine carbons were resonated at δ 56.84 (C-14), 55.75 (C-17), 42.54 (C-9), 36.15 (C-8), 36.06 (C-20), 29.87 (C-25) in addition to the three oxygenated methine carbons as mentioned above. There were also two quaternary carbon signals resonated at δ 42.20 (C-13) and 34.84 C-10) in addition to the quaternary carbon (C-5) of the epoxy group. Beside 5α,6α-epoxycholestan-3β-ol, its β-epoxy stereoisomer was also present, their ratios were (57:43). The main difference between them was observed for epoxy C signals which appeared at δ 62.91 (C-5) and 63.70 (C-6) for compound **6**, and proton chemical shift of the C-6 of compound **6** appeared at δ 3.06 (d, *J* = 2.3 Hz H-6α). The other noteworthy difference between two stereoisomers was observation of carbon and proton signals of C-3 which resonated at δ 69.41 and 3.69 (ttt, *J* = 10.0, 10.0, 4.9, 5.0) in compound **6**, respectively (Carvalho et al. 2009). The other signals appeared more or less at the same ppm’s. All ^1^H and ^13^C NMR (APT) signals of both compounds **5** and **6** can be seen in their NMR spectra given in the Supplementary Materials. Based on all spectral and literature data, structure of these two natural steroids, isolated from nature for the first time, were identified as 5α,6α-epoxycholestan-3β-ol (**5**) and 5β,6β-epoxycholestan-3β-ol (**6**).

A steroid ester, stigmast-5,22-dien-3β-ol myristate (**7**) has been recently isolated from *C. ovata* and published by our group (Sen et al. 2017) with anti-inflammatory and immunomodulatory actions which could be of interest for use in multiple sclerosis.

### Determination of anticholinesterase and anti-inflammatory activities

The anticholinesterase activity tests were carried out for all the five extracts prepared. The results of the anticholinesterase activity of the extracts were not noticeable against both AChE and BuChE enzymes (Table 3). The CHDFr extract showed weak-moderate activity at 200 µg/mL. However, CHDFl extract was found to be the most active extract against BuChE enzyme with 56.91 ± 0.99% inhibition at 200 µg/mL, CHDB showed the highest inhibition (20.226 ± 2.98%) against AChE (Table 3).

**Table 3.** Anticholinesterase activities of the extracts at 200 µg/mL^a^.

Samples	Inhibition of AChE (%)	Inhibition of BuChE (%)
CHDB	20.22 ± 2.98	25.78 ± 0.22
CHDFr	3.46 ± 0.05	NA
CHDFl	4.42 ± 1.42	56.91 ± 0.99
CHDL	8.12 ± 1.17	31.81 ± 2.31
CHDS	15.37 ± 3.02	NA
Galanthamine^b^	89.98 ± 0.61	82.47 ± 0.63

^a^Values expressed are means ± S.D. of 3 parallel measurements (*p* < 0.05). ^b^Standard drug. NA: Not active

Before anti-inflammatory and immunodulatory activity tests, the non-toxic doses of the secondary metabolites (**1–4**, **7**) were determined. In preclinical studies, EC_10_ (the lethal dose to 10% of cell treated) represents a safe phase I trial starting dose and EC values for human are best estimated for human SH-SY5Ycell lines. Therefore, the EC_10_ dose for tested pure compounds was investigated by crystal violet staining and the determined EC_10_ values on the expression levels of inflammatory genes involved in TNF pathway in SH-SY5Y cell lines that were given in Table 4.

**Table 4.** The effects of the pure secondary metabolites at the dose of EC_10_ on the expression levels of inflammatory genes involved in TNF pathway in SHSY-5Y cell line.

Expression Level*					
Gene	Comp. **1**	Comp. **2**	Comp. **3**	Comp. **4**	Comp. **7**
	(26 µM)	(14 µM)	(20 µM)	(25 µM)	(12 µM)
CCL5	–2.62	1.09	–2.43	–1.21	–1.68
CXCL9	–19.36	–2.58	–2.60	1.37	–1.91
CXCL10	–8.14	–1.67	–2.11	–1.46	–2.51
HIF1A	–6.26	1.74	–2.59	–1.06	–2.05
IL6	–2.22	–1.53	–2.39	–1.79	–1.82
NFKB	–4.52	1.79	–2.54	–1.05	–1.03
TNF-α	–18.69	–2.34	–4.49	–1.99	1.54

*The expression levels were given as fold changes as normalized relative to control. The positive values indicate increases and the negative values indicate the decreases. Cells shaded with light grey fillings shows significantly (*p* < 0.01) different values.

**Figure 2.**
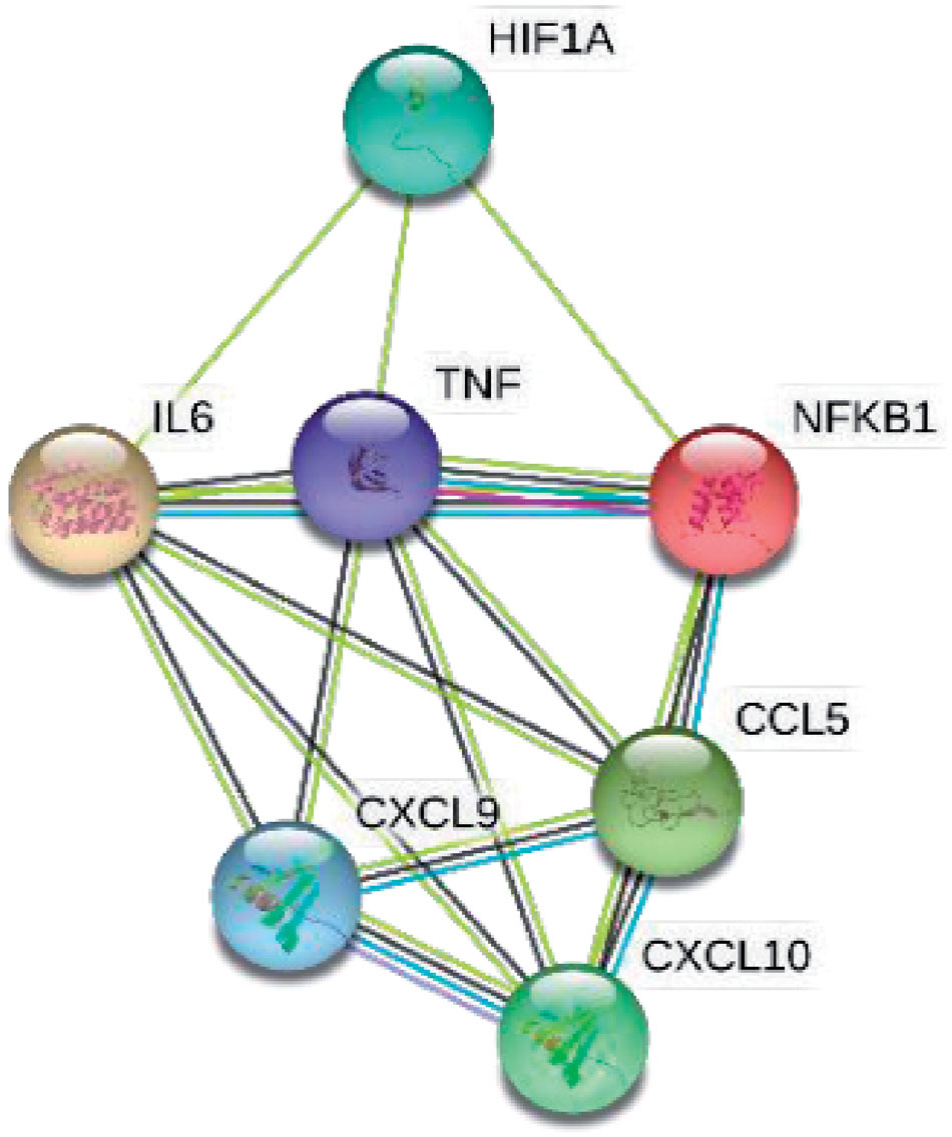
Protein-protein interaction network of the CCL5, CXCL9, CXCL10, HIF1A, IL6, NFKB1 and TNF-α genes visualized by STRING.

The effect of the purified secondary metabolites (PSM) on the expression of the selected genes participating in inflammation was determined in this study (Supplementary Table S1). The CCL5, CXCL9, CXCL10, HIF1A, IL6, NFKB1 and TNFα genes are known to be major players in the TNF signalling pathway. Their functional and physical association in this pathway were also depicted by STRING (Search Tool for the Retrieval of Interacting Genes/Proteins) analysis (Figure 2). TNF signalling plays a central role in inflammation and promotes inflammatory responses. The effect of the PSM on these genes was evaluated since caper is known to be utilized for its anti-inflammatory actions (Bektas et al. 2012). The new compound olean-12-en-28-ol, 3β-pentacosanoate (**1**) inhibited significantly the expression of all these inflammatory cytokines in SH-SY5Y cells, particularly CXCL9 and TNF-α. Both oleanolic acid (**2**) and its isomer ursolic acid (**3**) did inhibit the expression of TNF-α, additionally CXCL9 and CCL5. Moreover, ursolic acid inhibited nearly the same expression in all the genes tested. It has been shown that the agents blocking the TNF-α action may be used to treating problems associated with autoimmune diseases such as rheumatoid arthritis, inflammatory bowel disease and multiple sclerosis (Dinarello 2010).

## Conclusions

Triterpenoids and steroids were found to be the main isolates of the nonpolar extracts (hexane:dichloromethane) of the studied Anatolian *C. ovata* plant beside the fatty acids analysed by the GC-MS of the fruit extract (CHDFr) which showed that linoleic acid was the major fatty acid (30.90%) of the content as known unique essential omega-6 fatty acid for human. The new triterpenoid ester (**1**), ursolic acid (**2**), oleanolic acid (**3**) and a steroid ester stigmast-5,22-dien-3β-ol myristate (**7**) showed high anti-inflammatory activities. Among them, the new natural compound; olean-12-en-3β,28-diol, 3β-pentacosanoate (**1**) was found to be a promising candidate inhibiting significantly the expression of the tested inflammatory cytokines in SH-SY5Y cells, particularly CXCL9 and TNF-α. Thus, compound **1** should be considered as a potential therapeutic agent in the treatment of inflammatory and autoimmune diseases.

## Supplementary Material

Supplementary_Material.pdf
