# Supplementary material for: Triterpenoids and steroids isolated from Anatolian Capparis ovata and their activity on the expression of inflammatory cytokines
Source: Pharm Biol. 2020 Sep 11;58(1):925–31. doi: 10.1080/13880209.2020.1814356 (PMC7534286; doi:10.1080/13880209.2020.1814356)
Supplement: Supplementary_Material.pdf [file IPHB_A_1814356_SM5762.pdf]

SUPPLEMENTARY MATERIAL

Triterpenoids and steroids isolated from Anatolian *Capparis ovata* and their activity on the expression of inflammatory cytokines

Işıl Gazioğlu[a], Sevcan Şemen[b], Özden Özgün Acar[c], Ufuk Kolak[d], Alaattin Şen[c,e], Gülaçtı Topçu[f,*]

[a]Department of Analytical Chemistry, Faculty of Pharmacy, Bezmialem Vakif University, 34093 Fatih, Istanbul, Turkey

[b]Forensic Toxicology Laboratories, Institute of Forensic Sciences, Istanbul – Cerrahpasa University, 34098 Cerrahpasa, Istanbul, Turkey

[c]Department of Biology, Faculty of Arts and Sciences, Pamukkale University, 20070 Kinikli, Denizli, Turkey

[d]Department of Analytical Chemistry, Faculty of Pharmacy, Istanbul University, 34116 Beyazit, Istanbul, Turkey

[e]Department of Molecular Biology and Genetics, Faculty of Life & Natural Sciences, Abdullah Gül University, 38080 Kocasinan, Kayseri, Turkey

[f]Department of Pharmacognosy and Phytochemistry, Faculty of Pharmacy, Bezmialem Vakif University, 34093 Fatih, Istanbul, Turkey

***Olean-12-en-28-ol, 3β-pentacosanoate (3β-Pentacosanoylolean-12-en-28-ol)*(1): Amorphous, colourless, ^{1}H-NMR (600 MHz, $CDCl_3$): δ 5.19 (1H, t, *J*=2.5 Hz, H-12), 4.50 (1H, dd, *J*=5.5, 10.2 Hz, H-3), 3.55 (1H, d, *J*=10.5 Hz, H-28a), 3.22 (1H, d, *J*=10.5 Hz, H-28b), 2.30 (1H, t, *J*=7.35 Hz, H-2'), 1.66 (2H, hextet of doublet), 1.25 (3H, s, CH_3), 1.20 [m, $(CH_2)_n$], 1.17 (3H, s, CH_3), 0.96 (3H, s, CH_3), 0.94 (3H, s, CH_3), 0.89 (3H, s, CH_3), 0.87 (3H, s, CH_3), 0.86 (3H, t, end CH_3 of the long chain), 0.85 (3H, s, CH_3). ^{13}C NMR (150 MHz, $CDCl_3$): δ 38.1 (C-1), 23.4 (C-2), 81.0 (C-3), 37.6 (C-4), 55.1 (C-5), 18.1 (C-6), 32.4 (C-7), 39.8 (C-8), 49.0 (C-9), 36.8 (C-10), 23.4 (C-11), 122.1 (C-12), 144.1 (C-13), 41.6 (C-14), 25.1 (C-15), 25.2 (C-16), 31.8 (C-17), 42.2 (C-18), 46.3 (C-19), 34.0 (C-20), 30.9 (C-21), 38.1 (C-22), 27.9 (C-23), 16.6 (C-24), 15.4 (C-25), 16.6 (C-26), 25.8 (C-27), 69.7 (C-28), 33.1 (C-29), 23.5 (C-30), 29.7 $(CH_2)_n$, 14.0 (end CH_3), 173.6 (-COO-). MS (*m/z*) (rel. int.): 803.4 (6) $[M]^+$ $C_{55}H_{98}O_3$, 413.2 (29), 391.2 (8),126.9 (20), 84.9 (38), 83.0 (16), 71.0 (11).**

***5α,6α-Epoxycholestan-3β-ol* (5): Amorphous, colourless, ^{1}H-NMR (600 MHz, $CDCl_3$): δ 3.92 (1H, m, H-3α), 2.90 (1H, d, *J*=4.7 Hz, H-6β), 0.61 (3H, s, Me-18), 1.06 (3H, s, Me-19), 0.89 (3H, d, *J*=6.5 Hz, Me-21), 0.82 (3H, d, *J*=6.6 Hz, Me-26), 0.84 (3H, d, *J*=6.6 Hz, Me-27). ^{13}C-NMR (150 MHz, $CDCl_3$): δ 32.38 (C-1), 30.69 (C-2), 68.75 (C-3), 41.89 (C-4), 65.63 (C-5), 67.63 (C-6), 35.50 (C-7), 30.11 (C-8), 55.40 (C-9), 34.84 (C-10), 21.15 (C-11), 39.84 (C-12), 42.32 (C-13), 56.11 (C-14), 23.04 (C-15), 28.81 (C-16), 56.84 (C-17), 11.95 (C-18), 19.14 (C-19), 36.14 (C-20), 18.69 (C-21), 33.89 (C-22), 26.07 (C-23), 24.09 (C-24), 29.12 (C-25), 19.80 (C-26), 19.00 (C-27).**

***5β,6β-Epoxycholestan-3β-ol* (6):Amorphous, colourless, ^{1}H-NMR (600 MHz, $CDCl_3$): δ 3.70 (1H, m, H-3α), 3.06 (1H, d, *J*=2.3 Hz, H-6α), 0.64 (3H, s, Me-18),**

0.99 (3H, s, Me-19), 0.89 (3H, d, *J*= 6.5 Hz, Me-21), 0.82 (3H, d, *J=6.6* Hz, Me-26), 0.84 (3H, d, *J*=6.6 Hz, Me-27). ^{13}C-NMR (150 MHz, $CDCl_3$): δ 32.59 (C-1), 31.07 (C-2), 69.41 (C-3), 42.02 (C-4), 65.68 (C-5), 63.70 (C-6), 37.21 (C-7), 31.02 (C-8), 51.22 (C-9), 33.87 (C-10), 21.97 (C-11), 39.38 (C-12), 42.26 (C-13), 55.75 (C-14), 24.04 (C-15), 28.79 (C-16), 59.28 (C-17), 11.73 (C-18), 18.68 (C-19), 36.06 (C-20), 17.03 (C-21), 34.84 (C-22), 26.02 (C-23), 24.17 (C-24), 29.75 (C-25), 19.79 (C-26), 18.95 (C-27).

***Stigmast-5,22-dien-3β-olmyristate* (7): Amorphous, white,^{1}H-NMR (600 MHz, $CDCl_3$): δ 4.60 (1H, m, H-3α), 5.34 (1H, brd, *J*=4.1 Hz, H-6), 5.02 (m, H-23), 5.75 (m, H-22), 2.26 (dd, *J*= 2.7 Hz, H-4α), 2.31 (dd, *J*=6.5 Hz, H-4β), 0.68 (3H, s, Me-18), 1.02 (3H, s, Me-19), 0.92 (3H, d, *J*=6.5 Hz, Me-21), 0.81 (d, *J*=7.9 Hz, CH_3-27), 0.83 (d, *J*=6.8 Hz, CH_3-29), 1.25 (s, CH_3-28).0.83 (3H, d, *J*=6.8 Hz, Me-26), 0.85 (3H, t, *J*=7.1 Hz, Me-29).^{13}C-NMR (150 MHz, $CDCl_3$): δ 173.28 (C-1′), 36.98 (C-1), 31.90 (C-2), 73.67 (C-3), 42.28 (C-4), 139.69 (C-5), 122.56 (C-6), 31.88 (C-7), 36.13 (C-8), 51.42 (C-9), 36.57 (C-10), 21.01 (C-11), 39.69 (C-12), 42.29 (C-13), 56.67 (C-14), 24.27 (C-15), 28.23 (C-16), 56.01 (C-17), 11.95 (C-18), 19.00 (C-19), 45.81 (C-20), 20.18 (C-21), 130.03 (C-22), 128.02 (C-23), 49.99 (C-24), 31.90 (C-25), 21.00 (C-26), 19.30 (C-27), 29.33 (C-28), 11.83 (C-29), 29.3 $(CH_2)_n$. APCI-MS(m/z): 622.5 (4) [M^+] $C_{43}H_{74}O_2$, 607.5 (30), 448.2 (72), 381.2 (120), 365.2 (318), 349.2 (280), 333.2 (385).**

***Bis(7-methyloctyl)phythalate* (8): Amorphous, colourless.^{1}H-NMR (600 MHz, $CDCI_3$):δ 7.71 (2H, dd, *J*=7.0, *J*=3.5 Hz), 7.53 (2H, dd, *J*=7.0, *J*=3.5 Hz), 4.22 [4H (next to carbonyl groups), octet, *J*=6.0 Hz)], 1.68 (2H, m), 1.42-1.30 (10H, m), 0.93 (6H, d, *J*=7.6 Hz), 0.90 (6H, d, *J*=7.1).^{13}C-NMR (150 MHz, $CDCl_3$): δ167.73 (C=O), 132.42 (aromatic ring C), 130.85 (aromatic ring C), 128.77 (aromatic ring**

C), 68.11 (C-1), 38.70 (C-2), 30.33 (C-3), 29.68 (C-4), 28.90 (C-5), 23.72 (C-6), 22.96 (C-7), 14.09 (C-8), 10.93 (C-9).

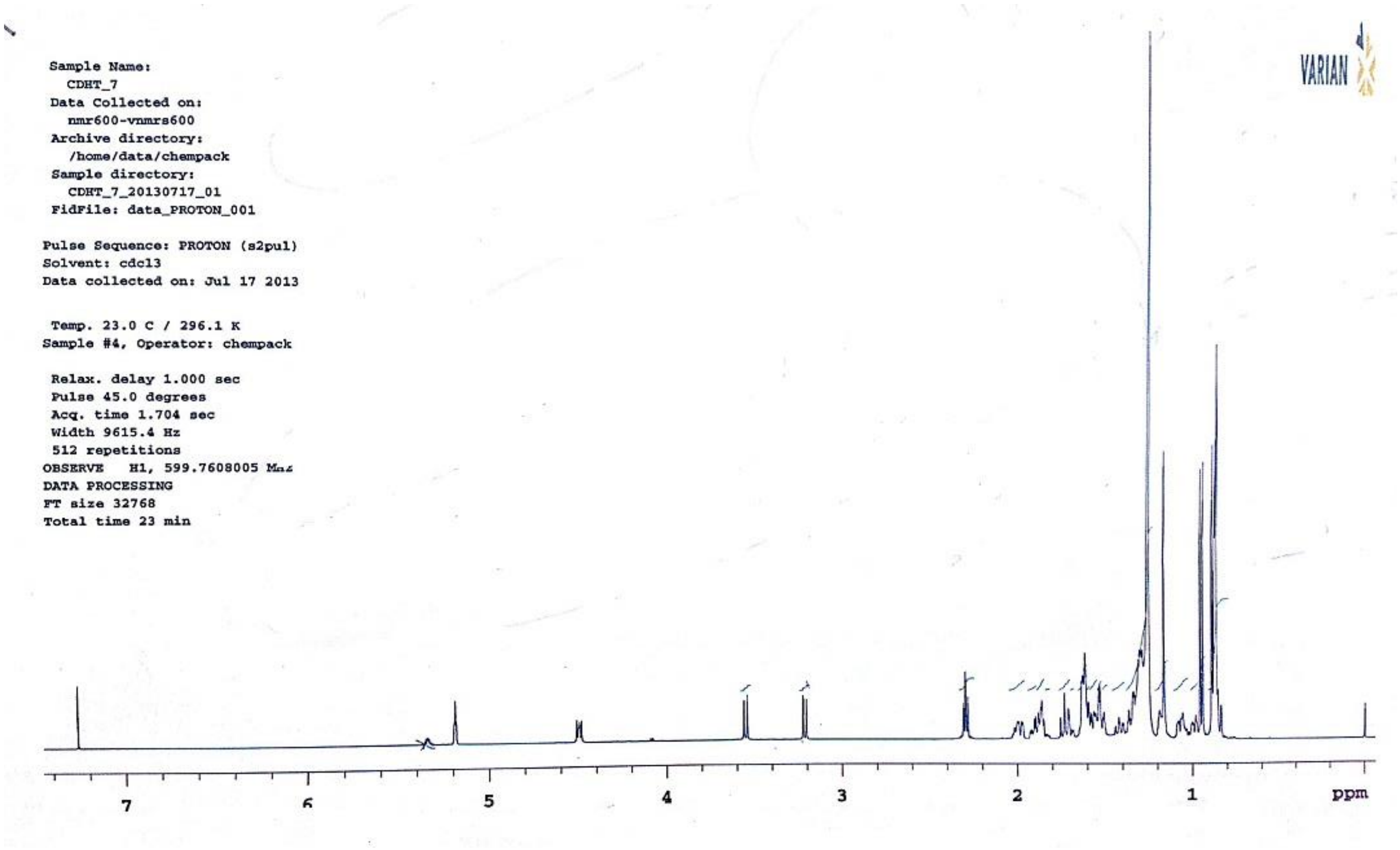

Fig 1. ^{1}H-NMR spectrum of comp. 3 (CDCI$_3$, 600 MHz, 0-7.4 ppm)

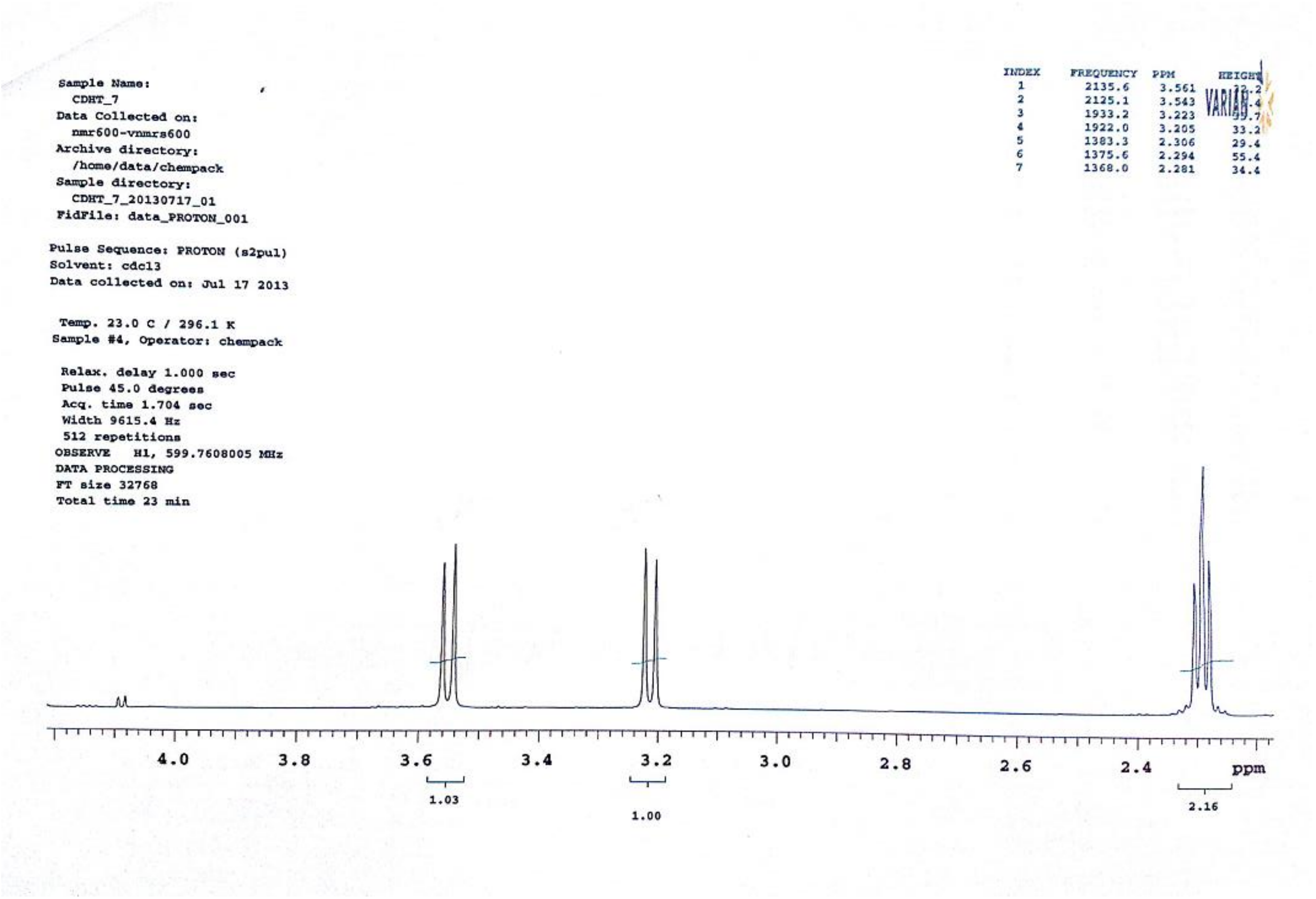

Fig. 2. ^{1}H-NMR spectrum of comp. 3 ($CDCl_3$, 600 MHz, 2.2-4.2 ppm)

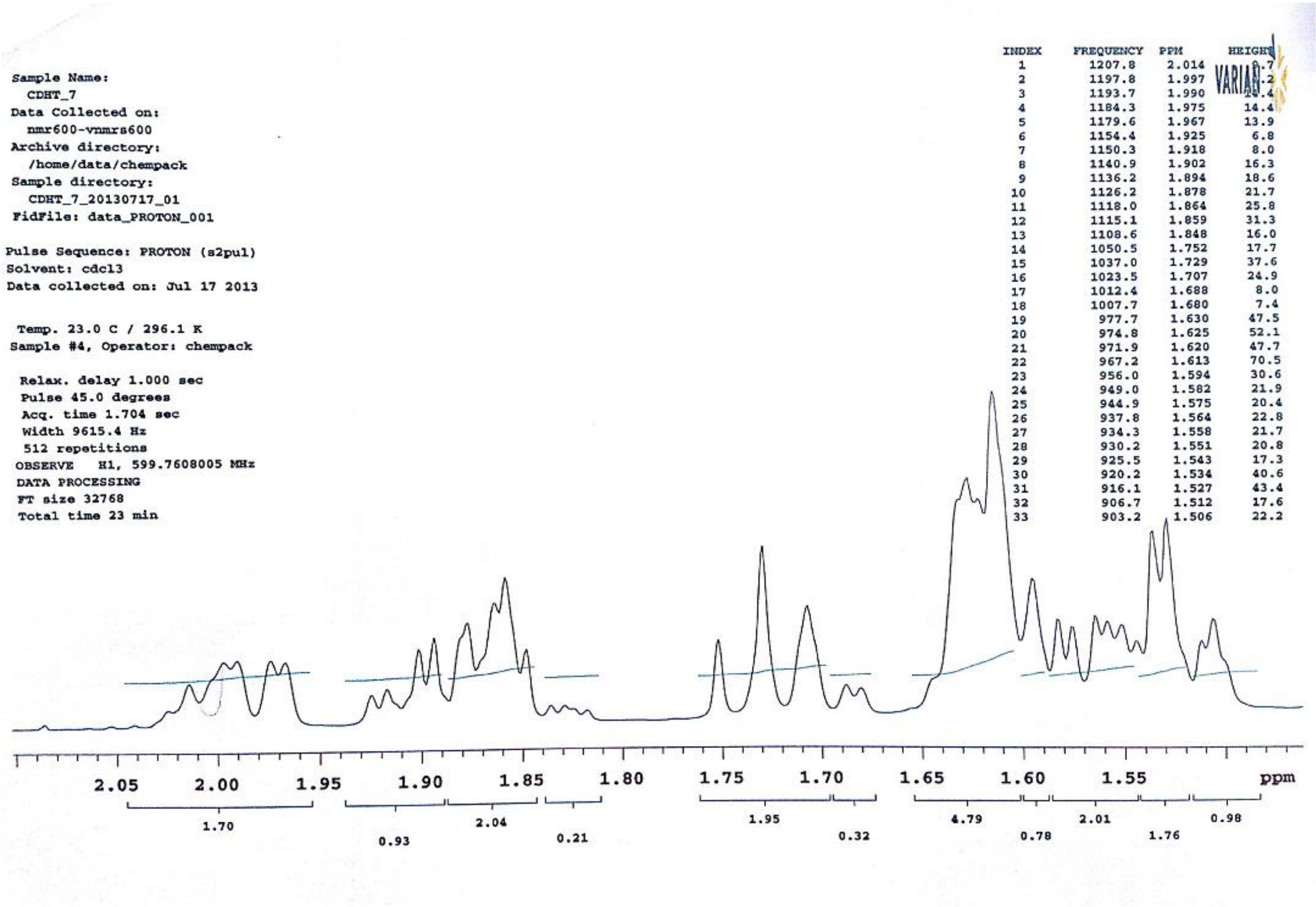

Fig. 3. ^{1}H-NMR spectrum of comp. 3 ($CDCI_3$, 600 MHz, 1.50-2.10 ppm)

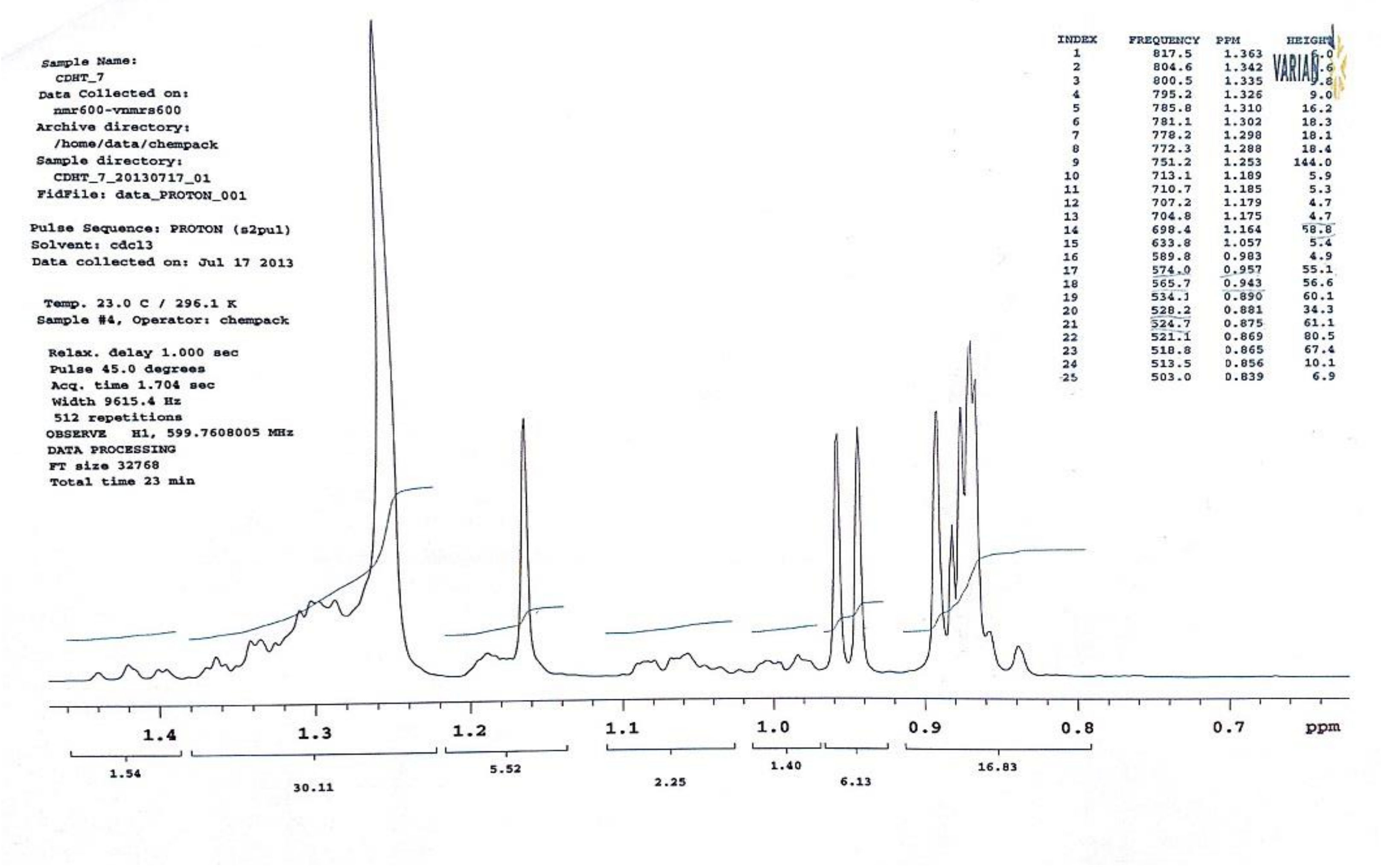

Fig. 4. ^{1}H-NMR spectrum of comp. 3 ($CDCl_3$, 600 MHz, 0.7-1.4 ppm)

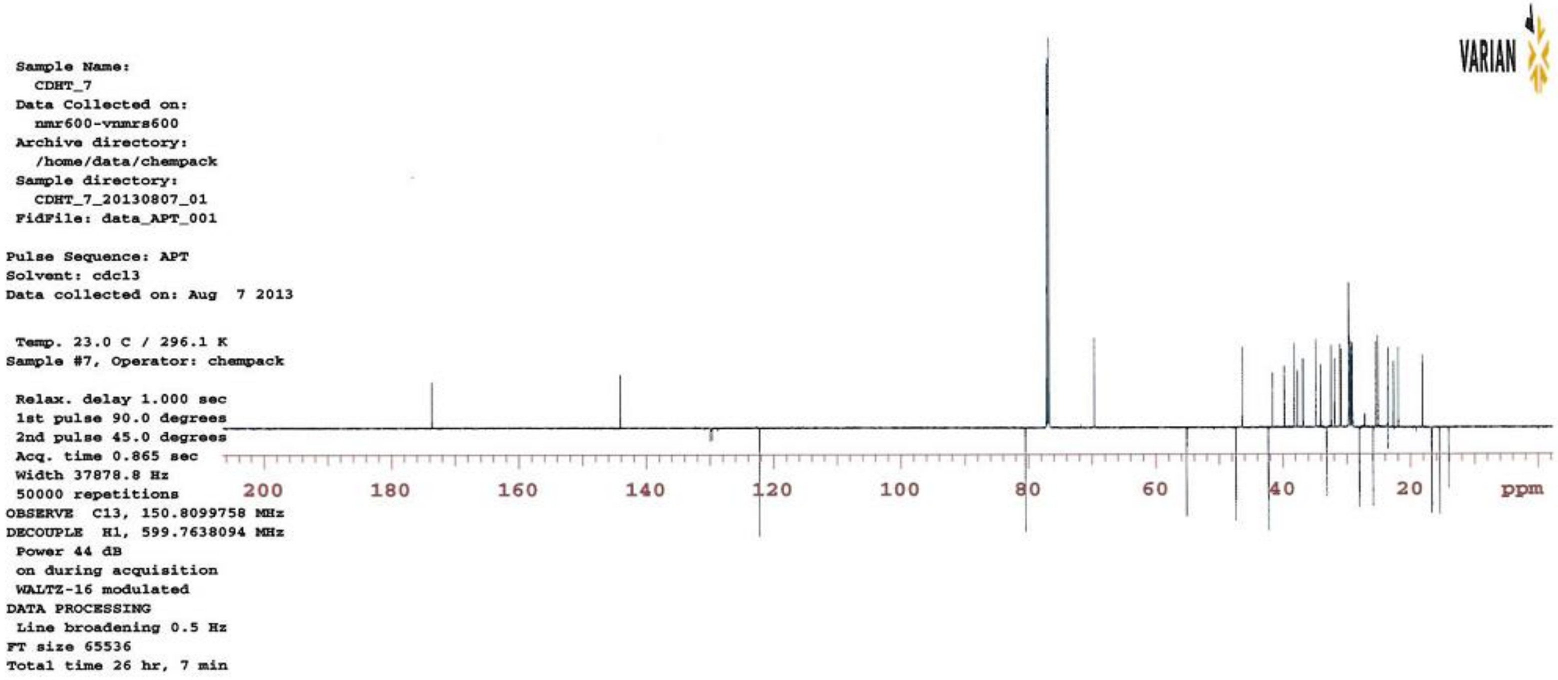

Fig. 5. ^{13}C-NMR spectrum of comp. 3 ($CDCl_3$, 125 MHz, 0-200 ppm)

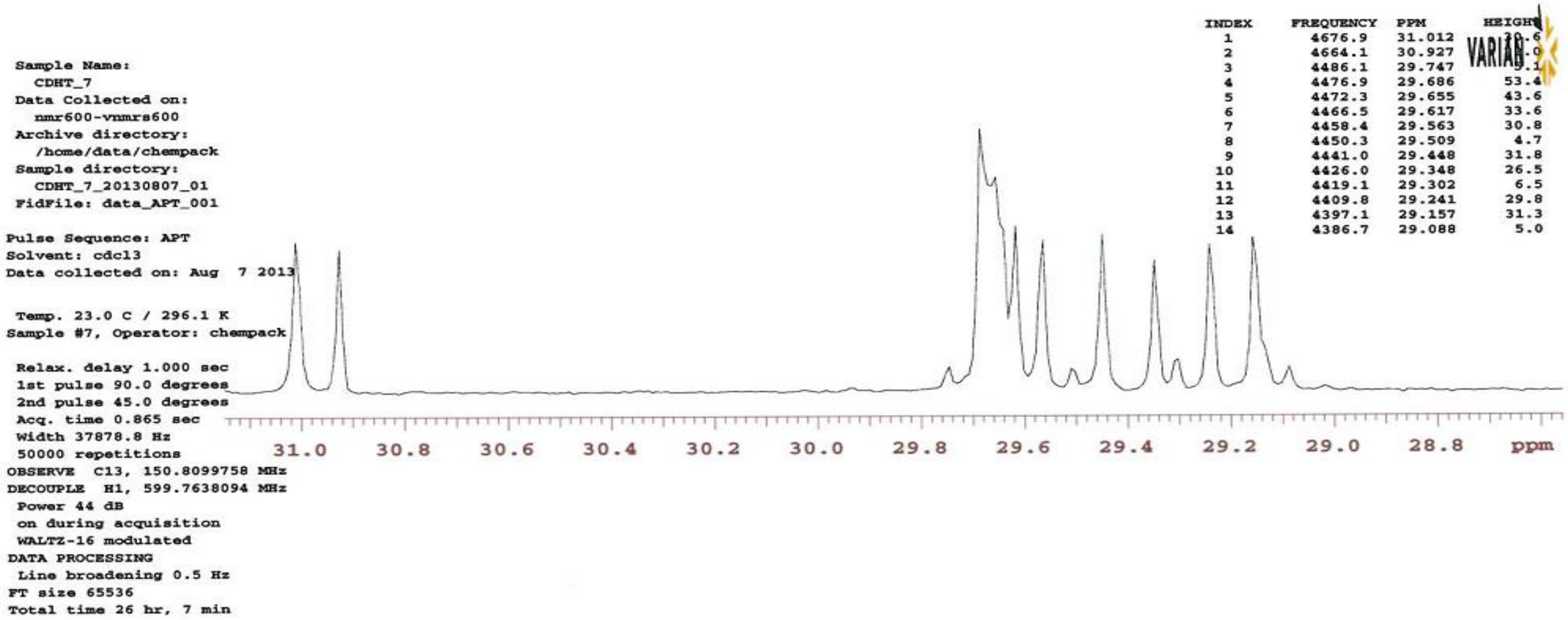

Fig. 6. ^{13}C-NMR spectrum of comp. 3 ($CDCl_3$, 125 MHz, 27-31 ppm)

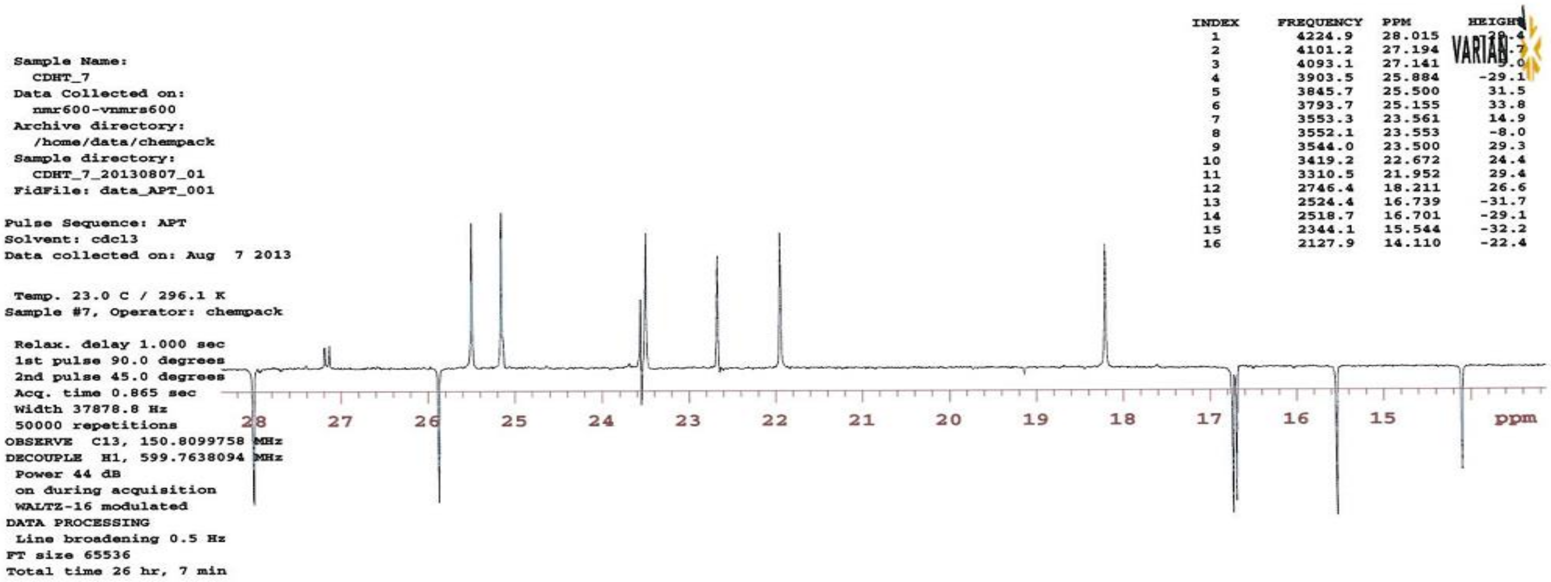

Fig. 7. ^{13}C-NMR spectrum of comp. 3 ($CDCI_3$, 125 MHz, 14-28 ppm)

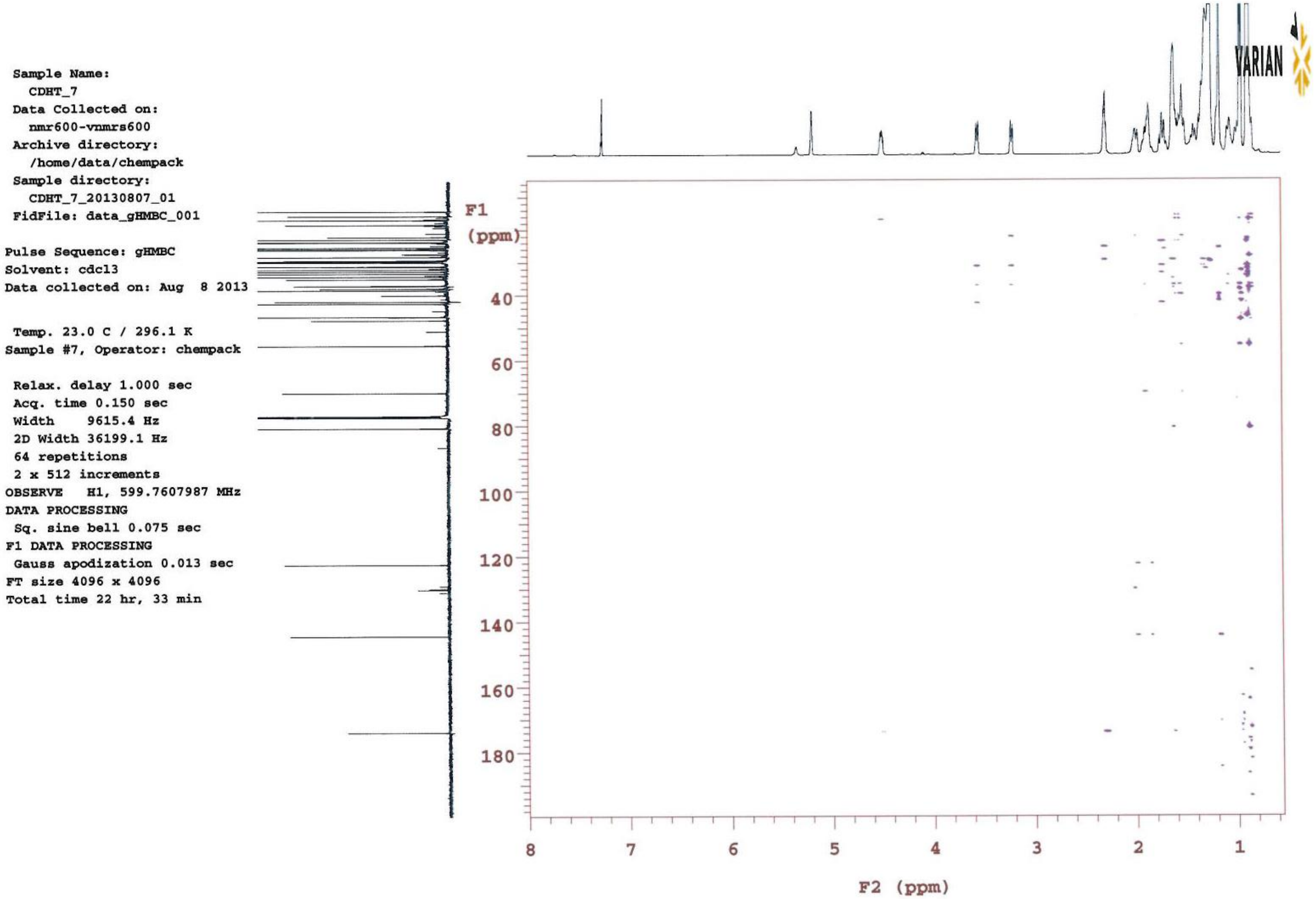

Fig. 8. HMBC spectrum of comp. 3 ($CDCl_3$, 125 MHz)

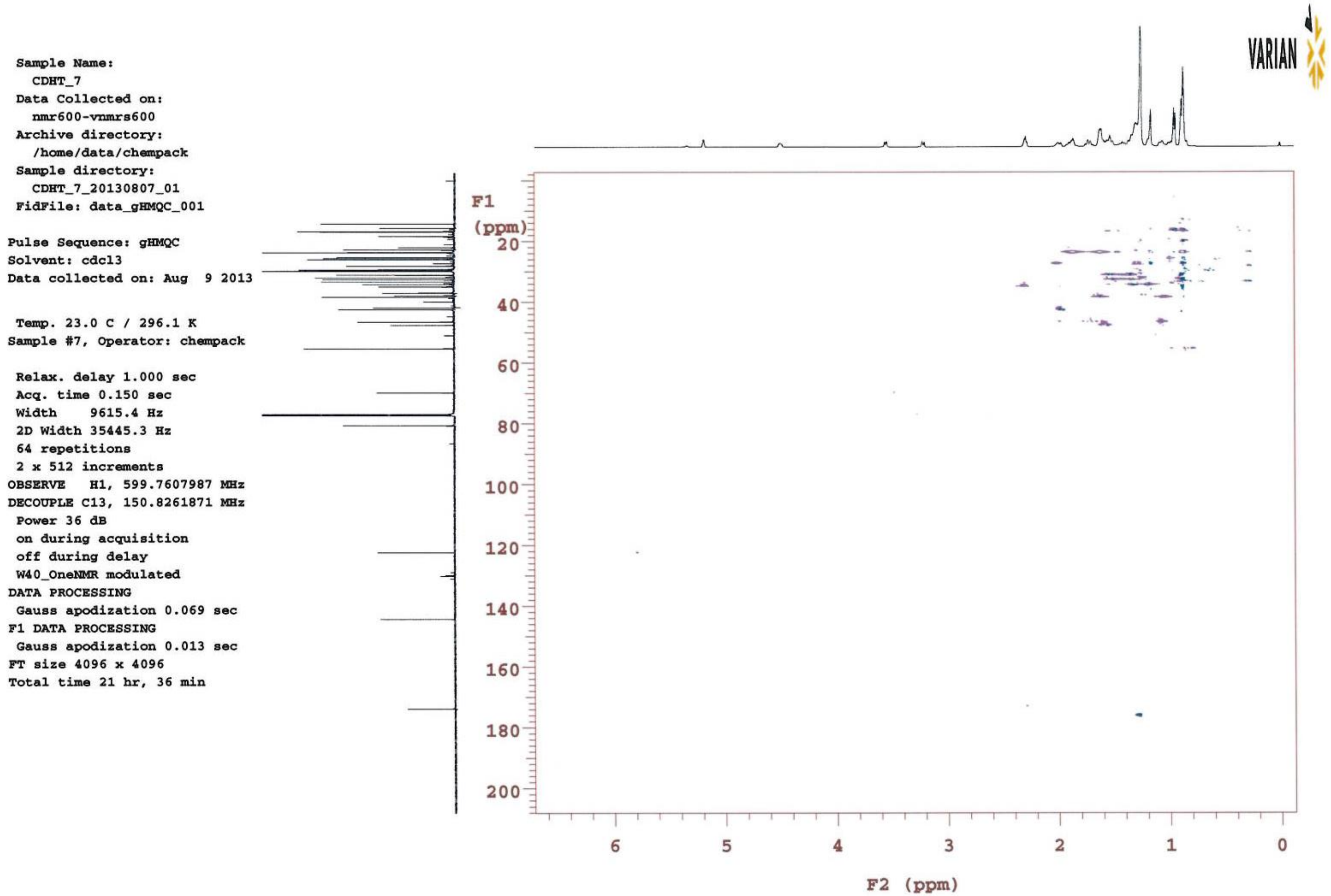

Fig. 9. HMQC spectrum of comp. 3 ($CDCl_3$, 125 MHz)

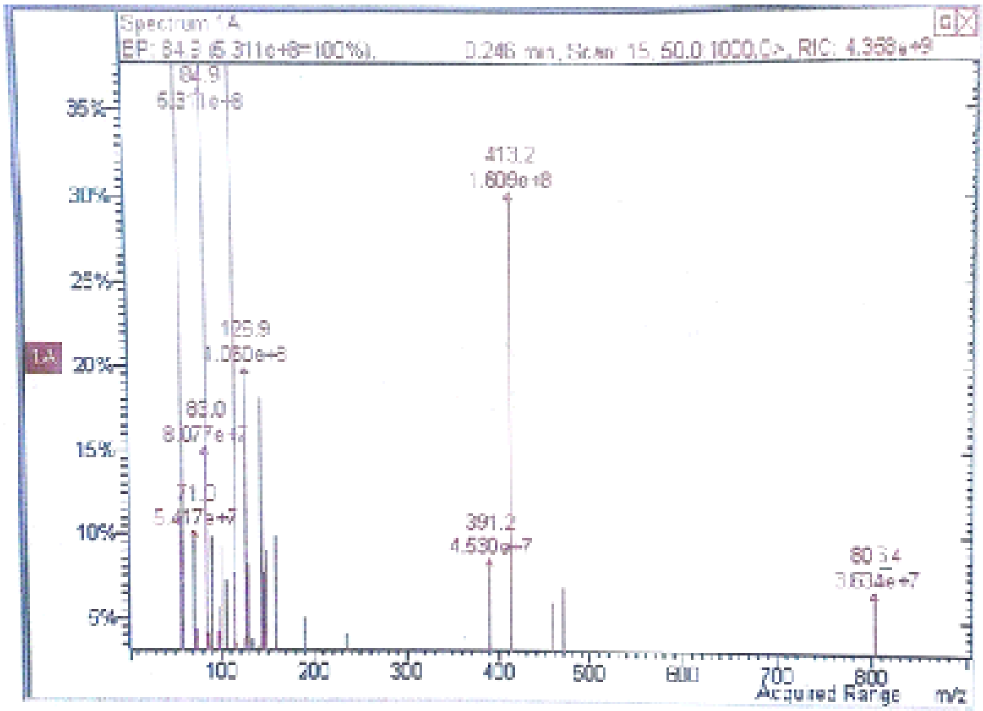

Fig. 10. MS spectrum of comp. 3

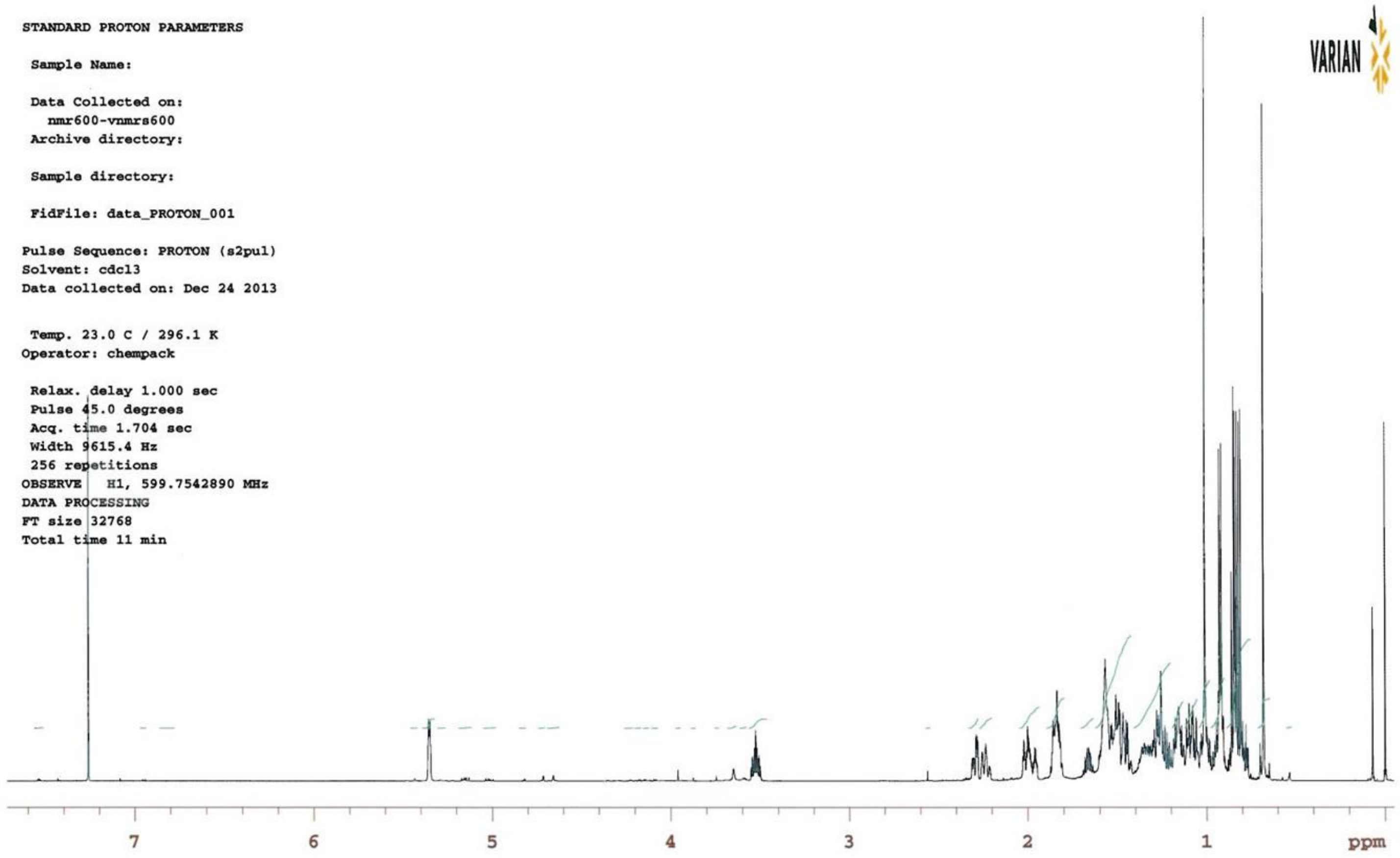

Fig. 11. ^{1}H-NMR spectrum of comp. 4 ($CDCl_3$, 600 MHz)

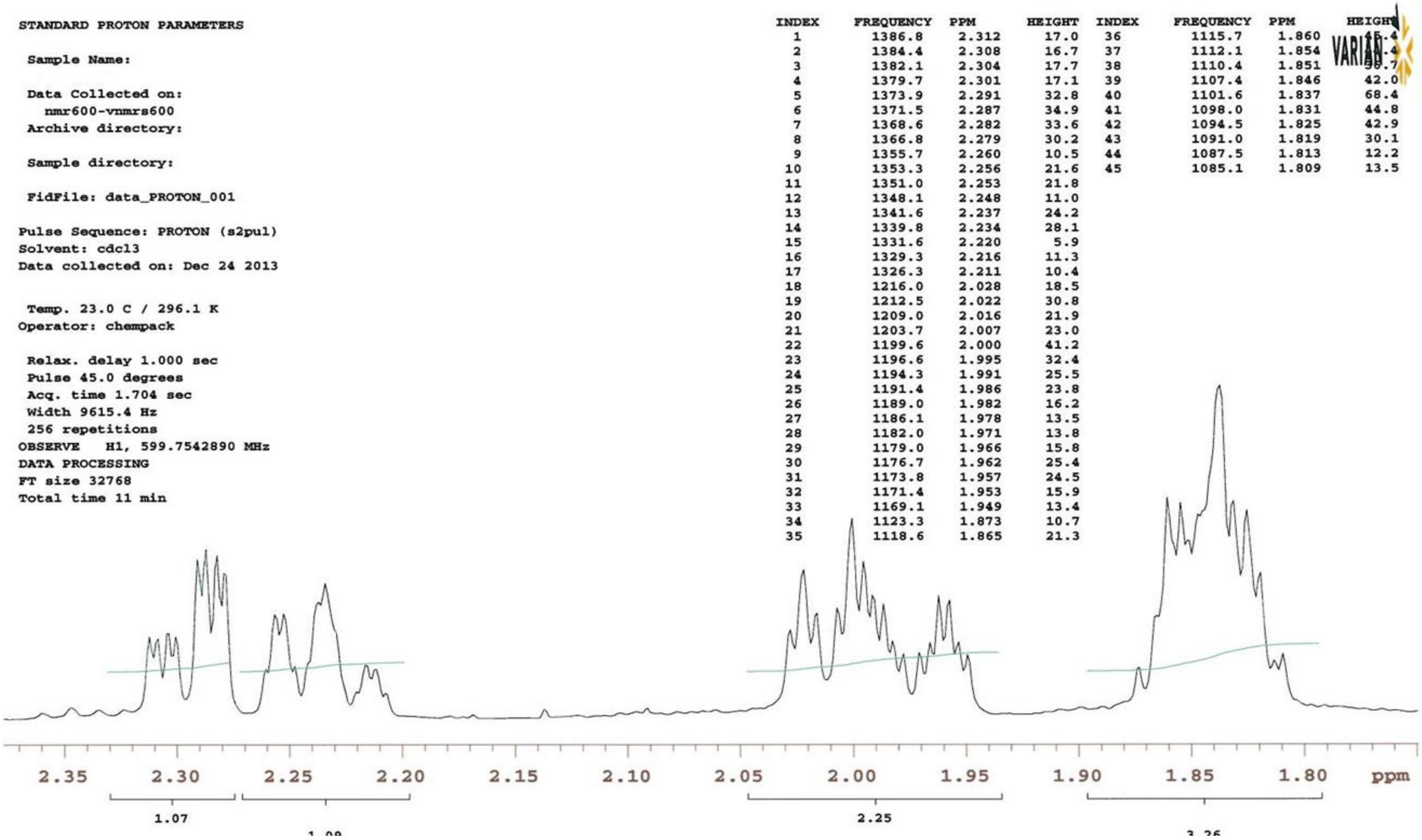

Fig. 12. ^{1}H-NMR spectrum of comp. 4 ($CDCl_3$, 600 MHz, 1.80-2.35 ppm)

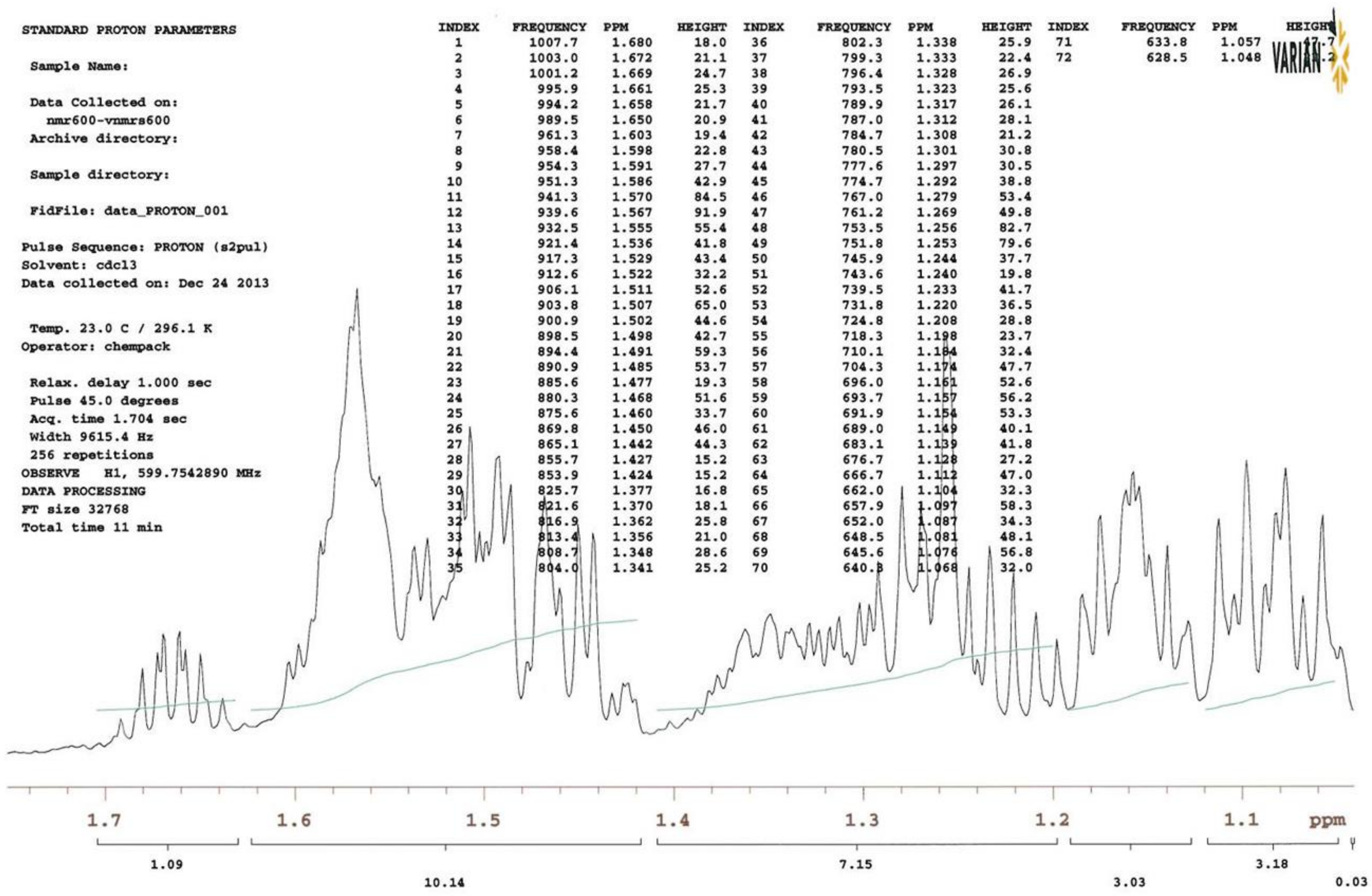

INDEX	FREQUENCY	PPM	HEIGHT
1	1007.7	1.680	18.0
2	1003.0	1.672	21.1
3	1001.2	1.669	24.7
4	995.9	1.661	25.3
5	994.2	1.658	21.7
6	989.5	1.650	20.9
7	961.3	1.603	19.4
8	958.4	1.598	22.8
9	954.3	1.591	27.7
10	951.3	1.586	42.9
11	941.3	1.570	84.5
12	939.6	1.567	91.9
13	932.5	1.555	55.4
14	921.4	1.536	41.8
15	917.3	1.529	43.4
16	912.6	1.522	32.2
17	906.1	1.511	52.6
18	903.8	1.507	65.0
19	900.9	1.502	44.6
20	898.5	1.498	42.7
21	894.4	1.491	59.3
22	890.9	1.485	53.7
23	885.6	1.477	19.3
24	880.3	1.468	51.6
25	875.6	1.460	33.7
26	869.8	1.450	46.0
27	865.1	1.442	44.3
28	855.7	1.427	15.2
29	853.9	1.424	15.2
30	825.7	1.377	16.8
31	821.6	1.370	18.1
32	816.9	1.362	25.8
33	813.4	1.356	21.0
34	808.7	1.348	28.6
35	804.0	1.341	25.2

INDEX	FREQUENCY	PPM	HEIGHT
36	802.3	1.338	25.9
37	799.3	1.333	22.4
38	796.4	1.328	26.9
39	793.5	1.323	25.6
40	789.9	1.317	26.1
41	787.0	1.312	28.1
42	784.7	1.308	21.2
43	780.5	1.301	30.8
44	777.6	1.297	30.5
45	774.7	1.292	38.8
46	767.0	1.279	53.4
47	761.2	1.269	49.8
48	753.5	1.256	82.7
49	751.8	1.253	79.6
50	745.9	1.244	37.7
51	743.6	1.240	19.8
52	739.5	1.233	41.7
53	731.8	1.220	36.5
54	724.8	1.208	28.8
55	718.3	1.198	23.7
56	710.1	1.184	32.4
57	704.3	1.174	47.7
58	696.0	1.161	52.6
59	693.7	1.157	56.2
60	691.9	1.154	53.3
61	689.0	1.149	40.1
62	683.1	1.139	41.8
63	676.7	1.128	27.2
64	666.7	1.112	47.0
65	662.0	1.104	32.3
66	657.9	1.097	58.3
67	652.0	1.087	34.3
68	648.5	1.081	48.1
69	645.6	1.076	56.8
70	640.8	1.068	32.0
71	633.8	1.057	[illegible]
72	628.5	1.048	[illegible]

Fig. 13. [1]**H-NMR spectrum of comp. 4 ($CDCl_3$, 600 MHz, 1.80-2.35 ppm)**

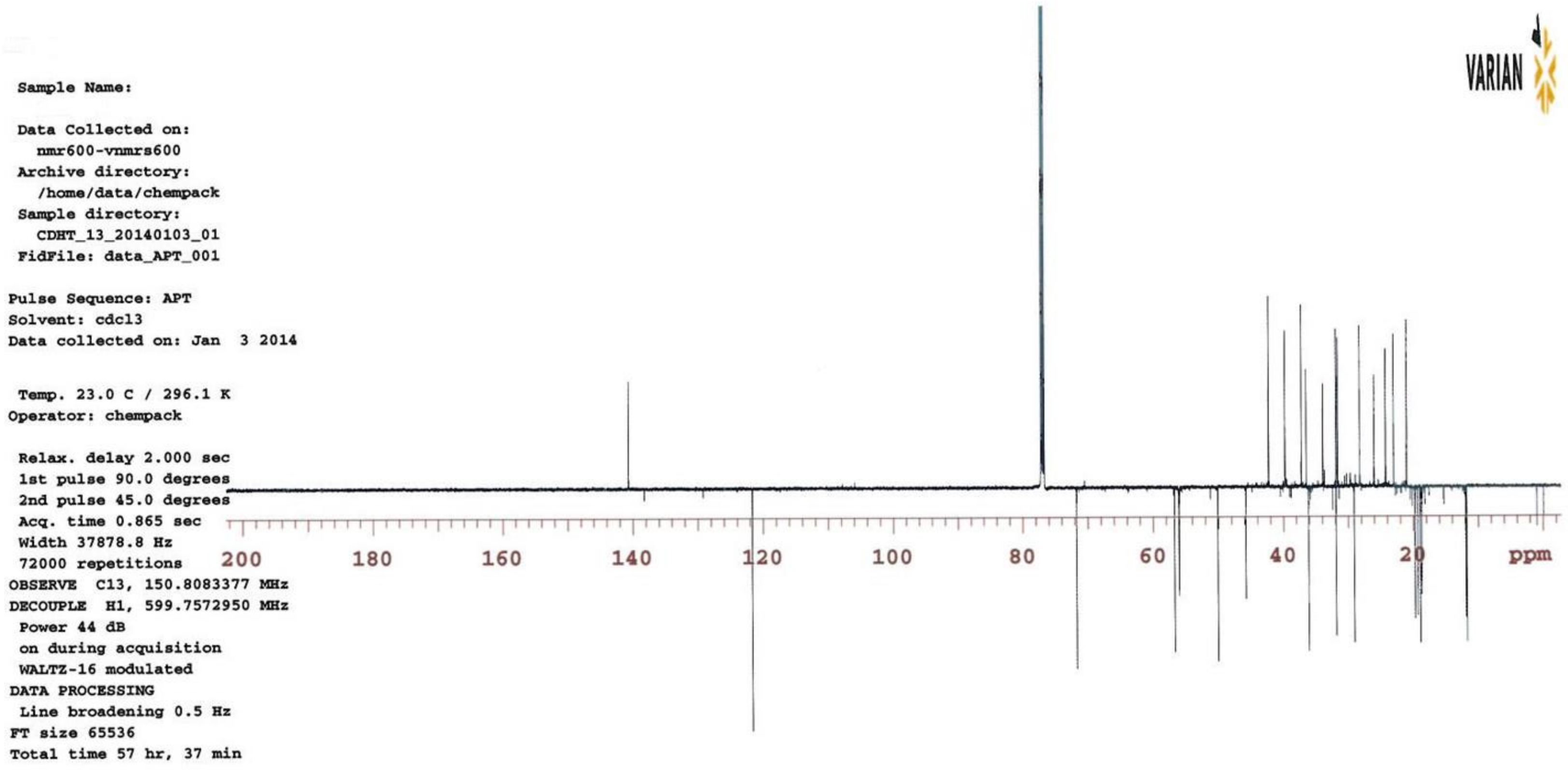

Fig. 14. ^{13}**C-NMR spectrum of comp. 4 (CDCl$_3$, 125 MHz, 10-200 ppm)**

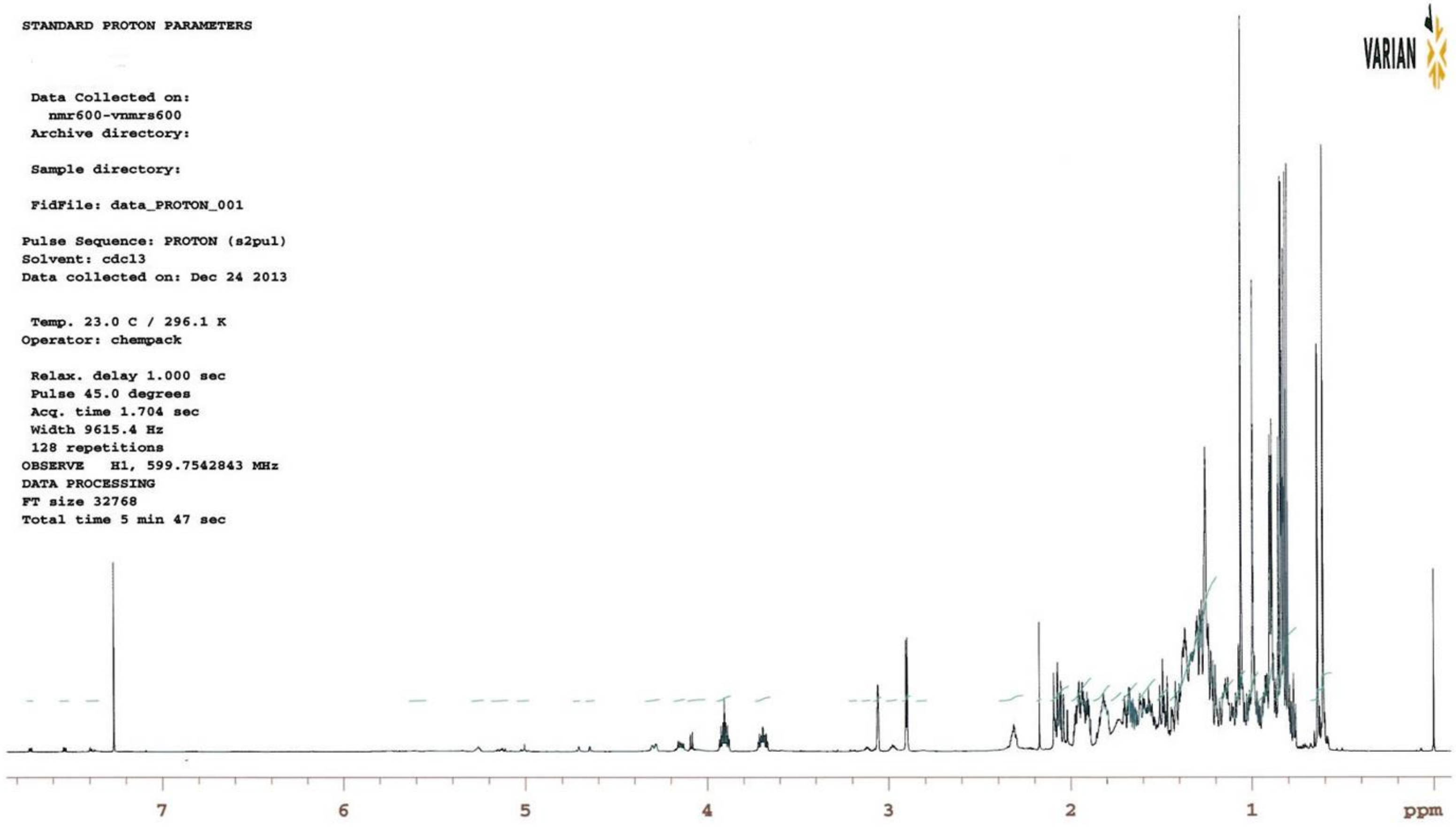

Fig. 15. ^{1}H -NMR spectrum of comp. 5-6 (CDCl$_3$, 600 MHz)

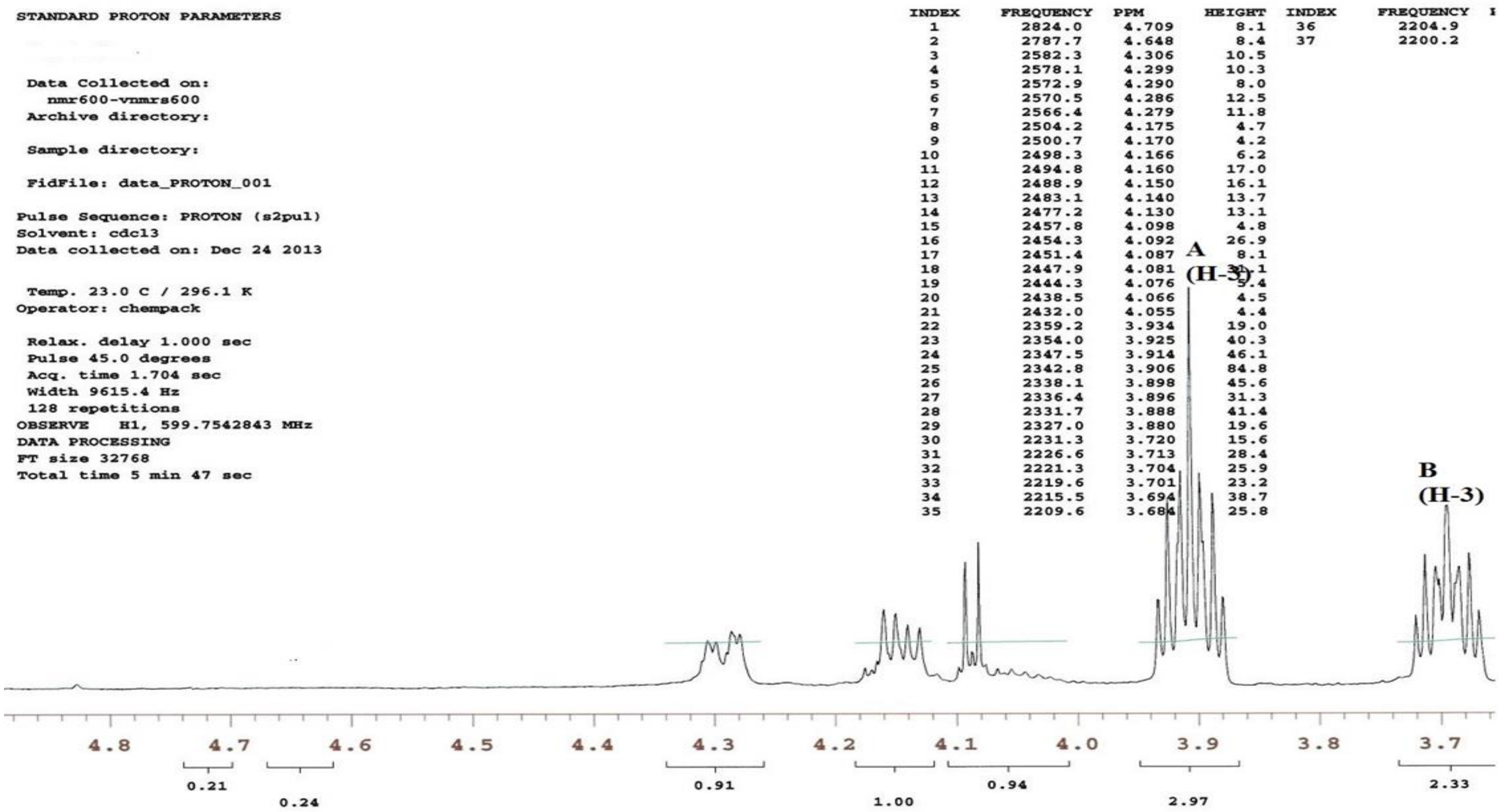

Fig. 16. ^{1}H -NMR spectrum of comp. 5-6 ($CDCl_3$, 600 MHz, 3.7-4.8 ppm)

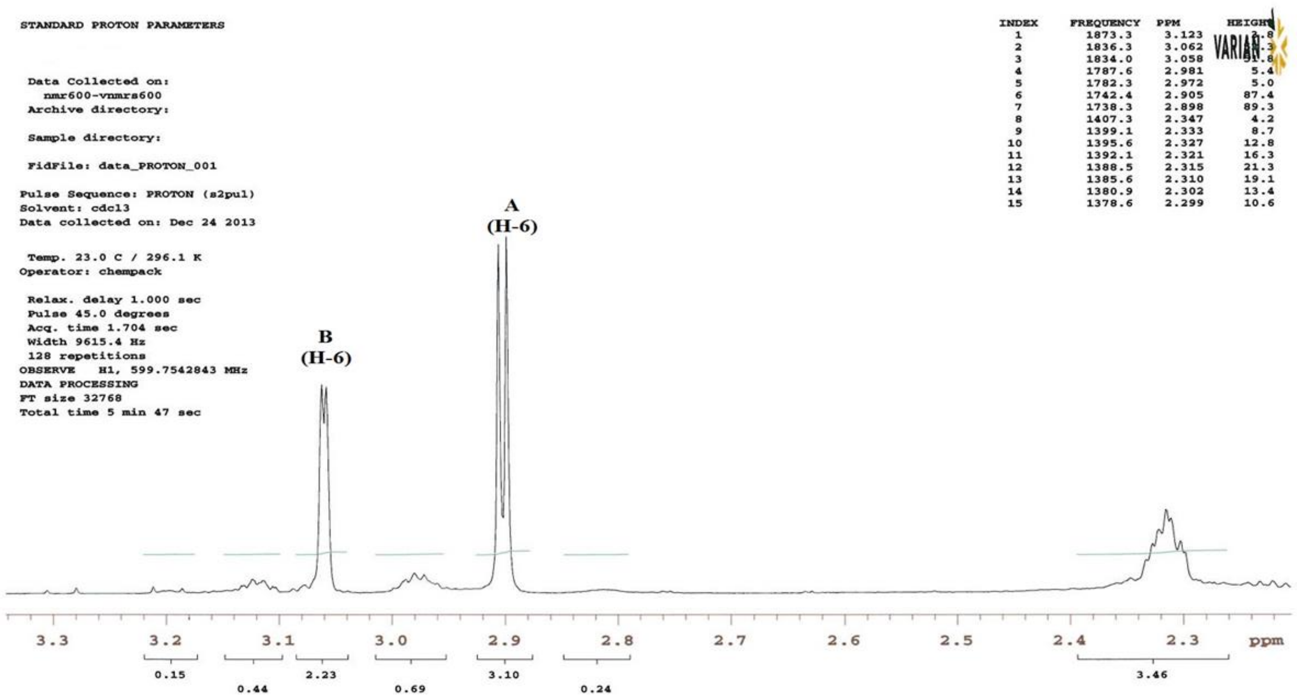

Fig. 17. ^{1}H -NMR spectrum of comp. 5-6 ($CDCl_3$, 600 MHz, 2.3-3.3 ppm)

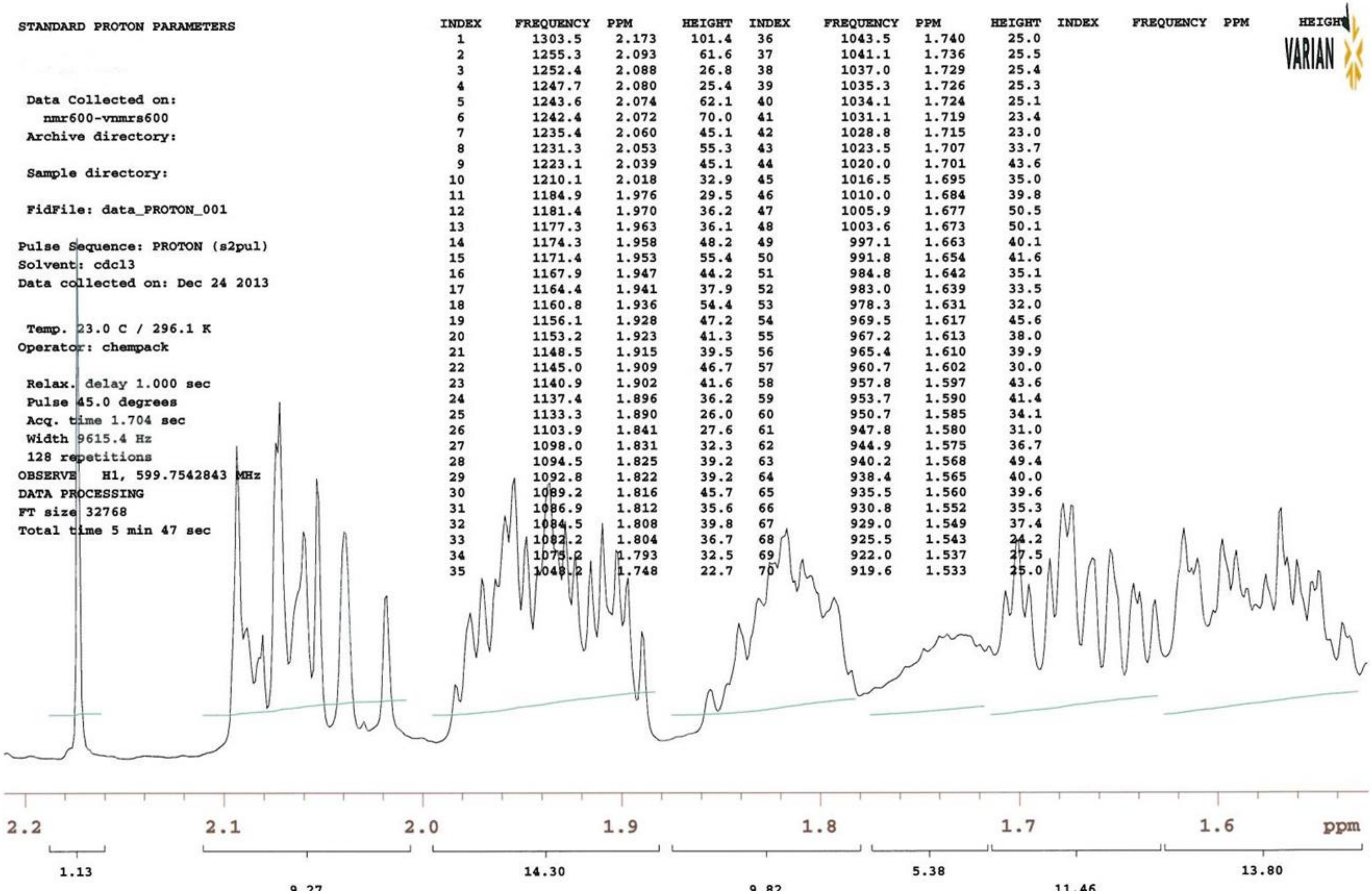

Fig. 18. ^{1}H-NMR spectrum of comp. 5-6 ($CDCl_3$, 600 MHz, 1.6-2.2 ppm)

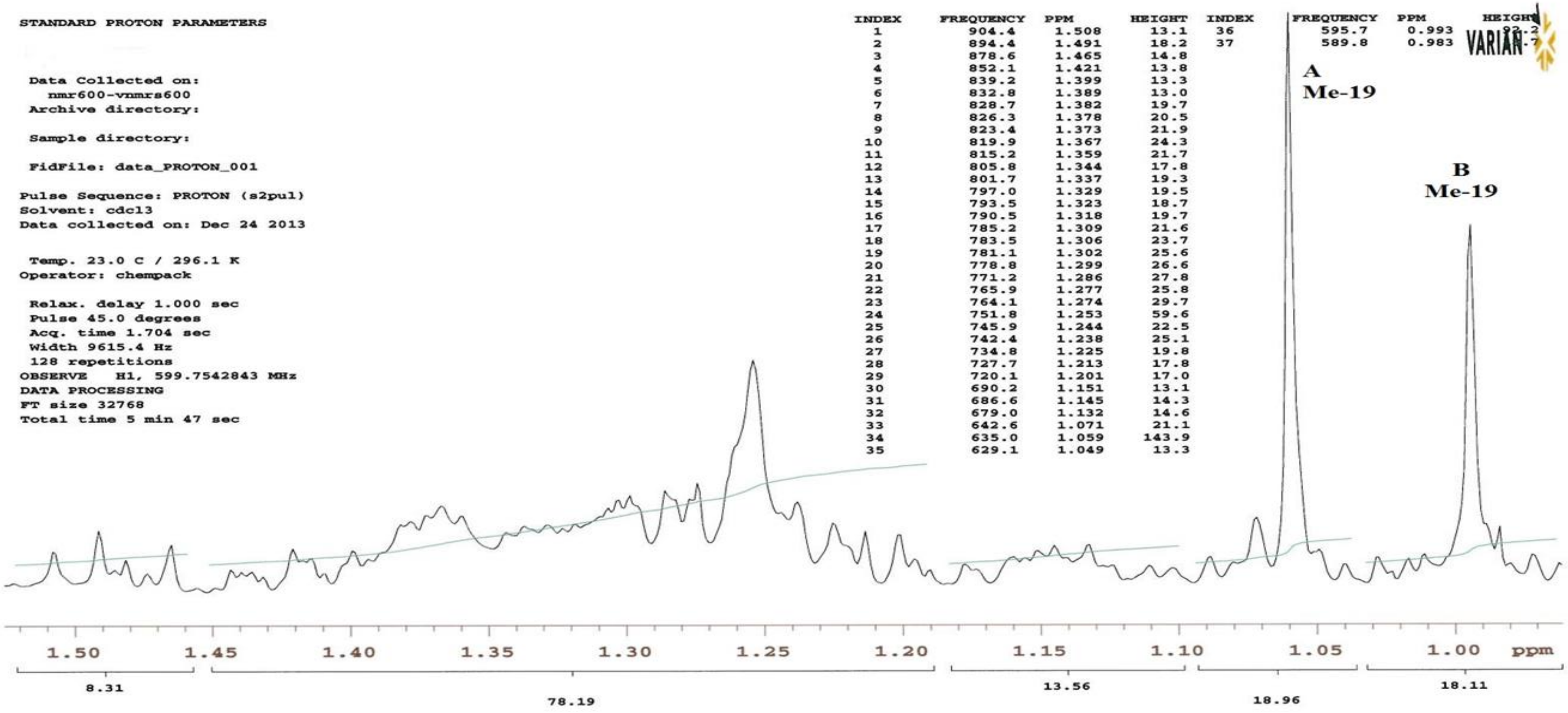

Fig. 19. **^{1}H-NMR spectrum of comp. 5-6 ($CDCl_3$, 600 MHz, 1.0-1.50 ppm)**

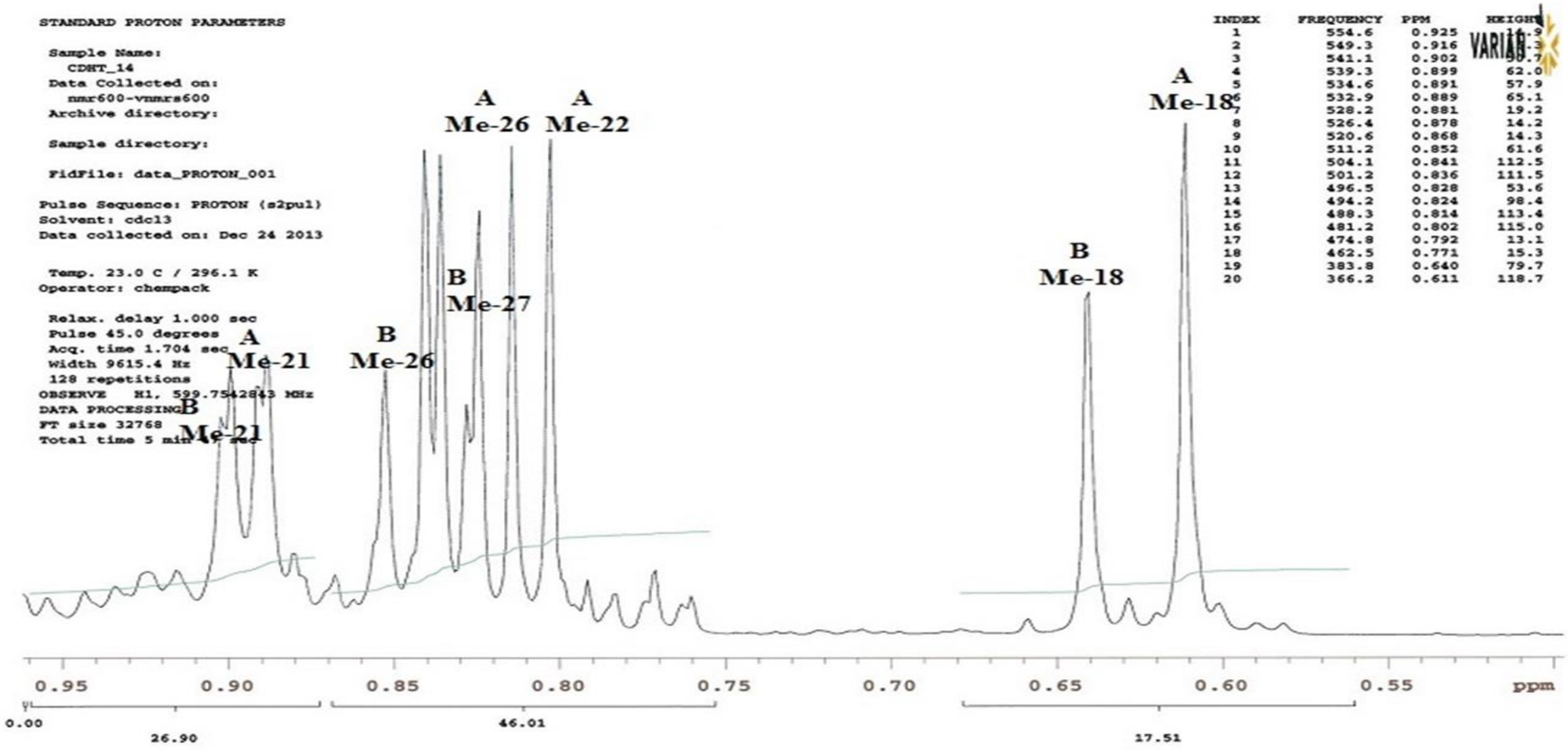

Fig. 19. ^{1}H-NMR spectrum of comp. 5-6 ($CDCl_3$, 600 MHz, 0.55-0.95 ppm)

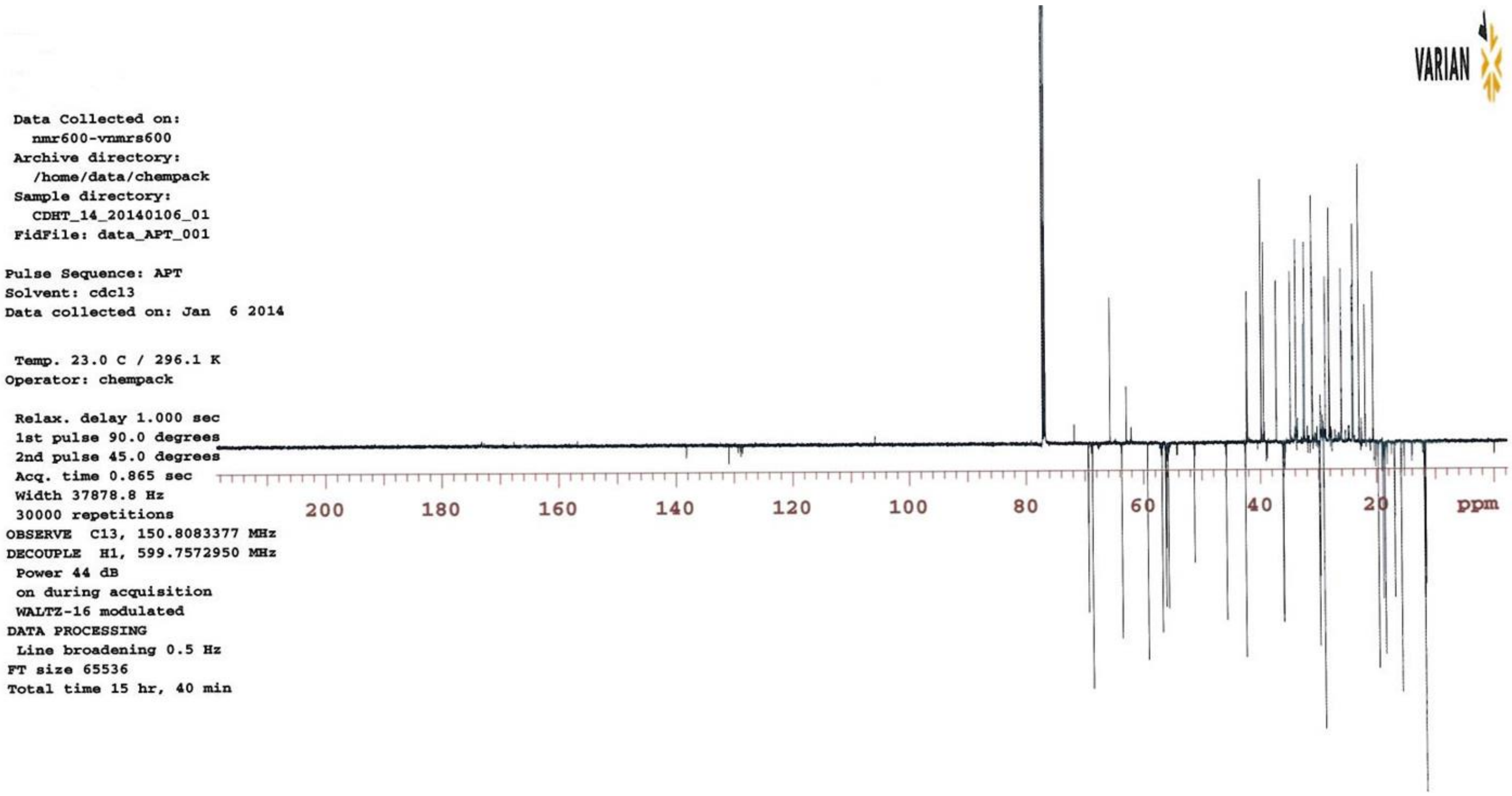

Fig. 20. ^{13}**C -NMR spectrum of comp. 5-6 (CDCI$_3$, 125 MHz)**

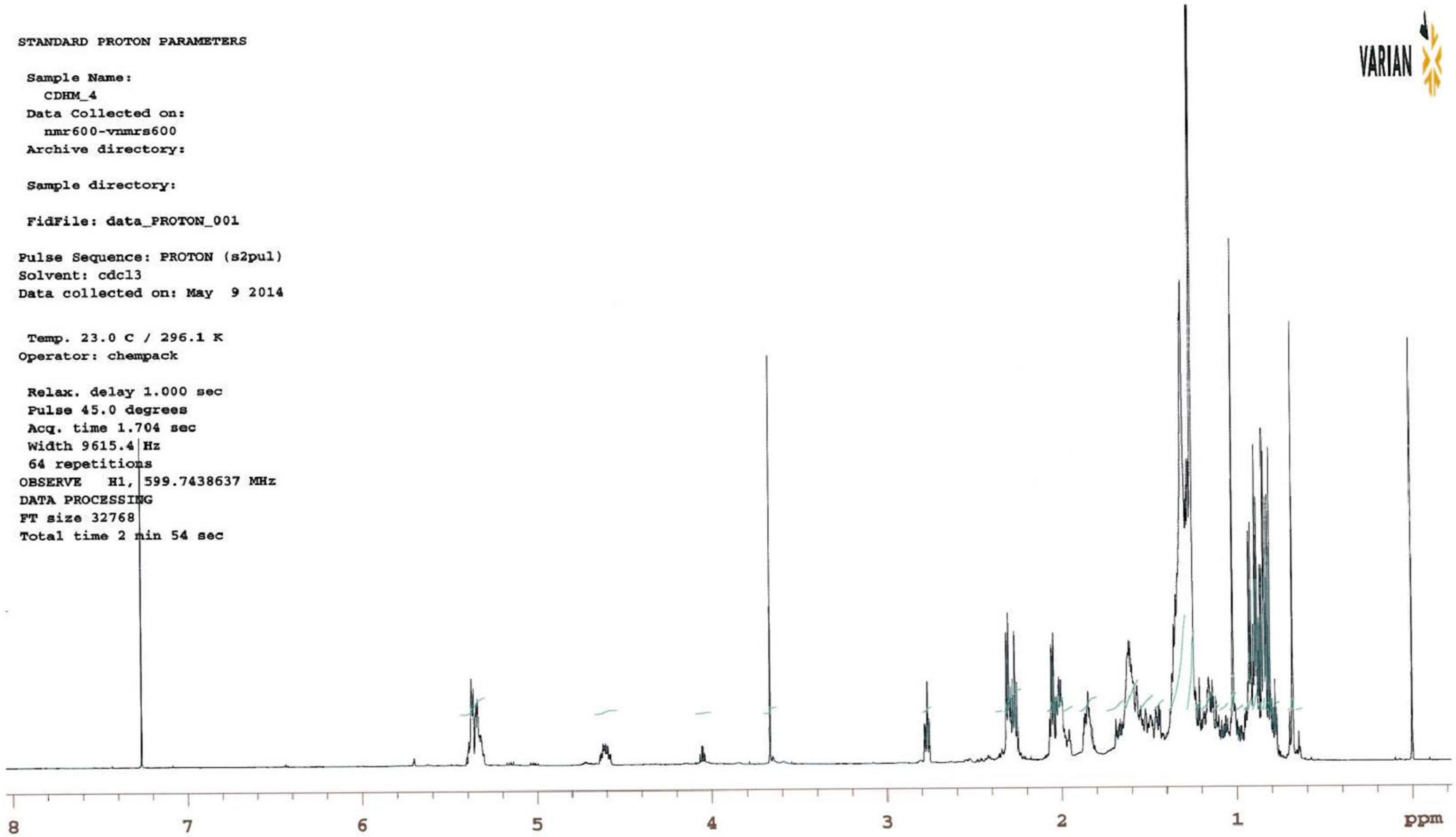

Fig. 21. ^{1}H-NMR spectrum of comp. 7 ($CDCl_3$, 600 MHz)

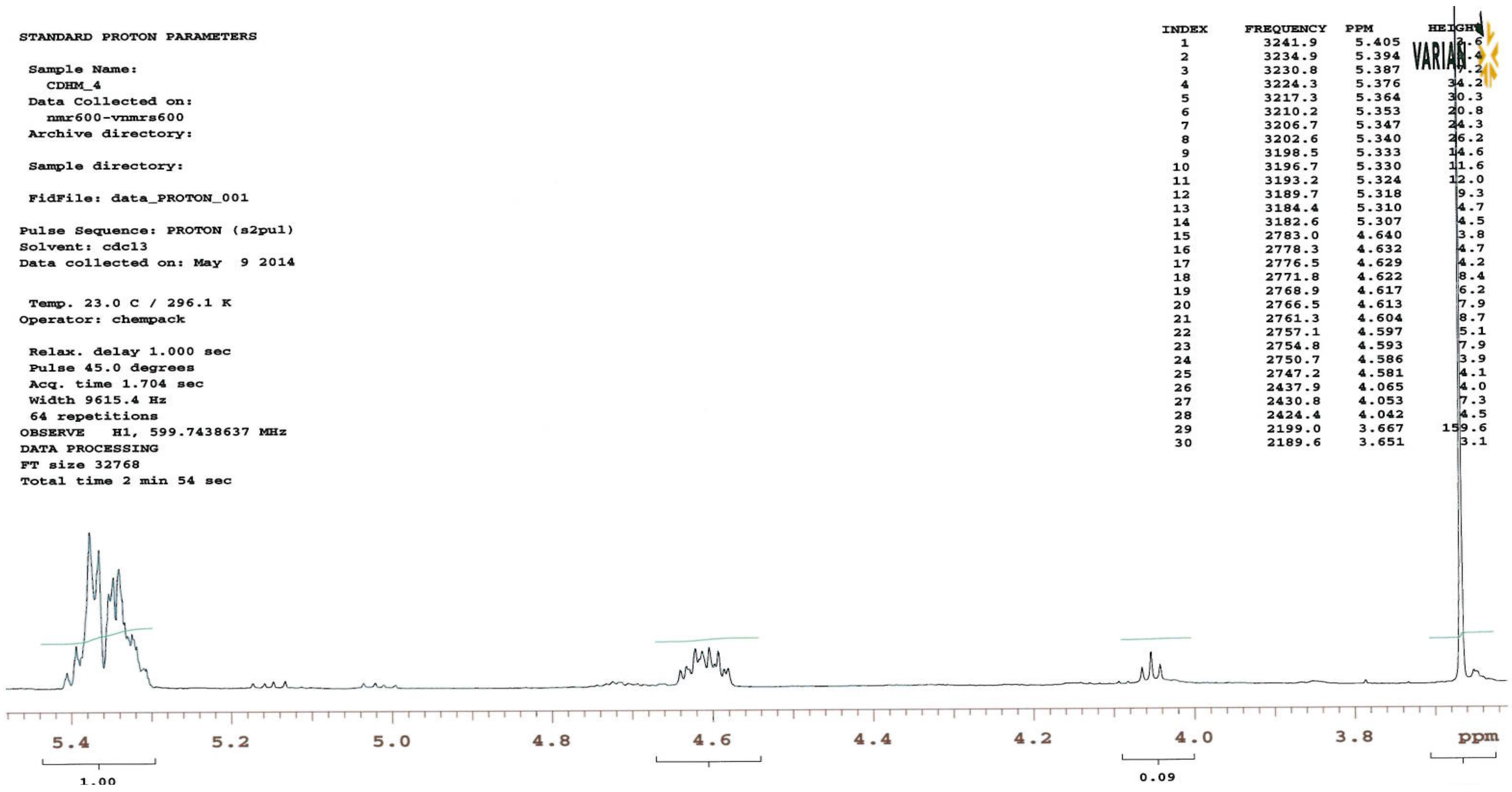

Fig. 22. ^{1}H-NMR spectrum of comp. 7 ($CDCl_3$, 600 MHz, 3.8-5.4 ppm)

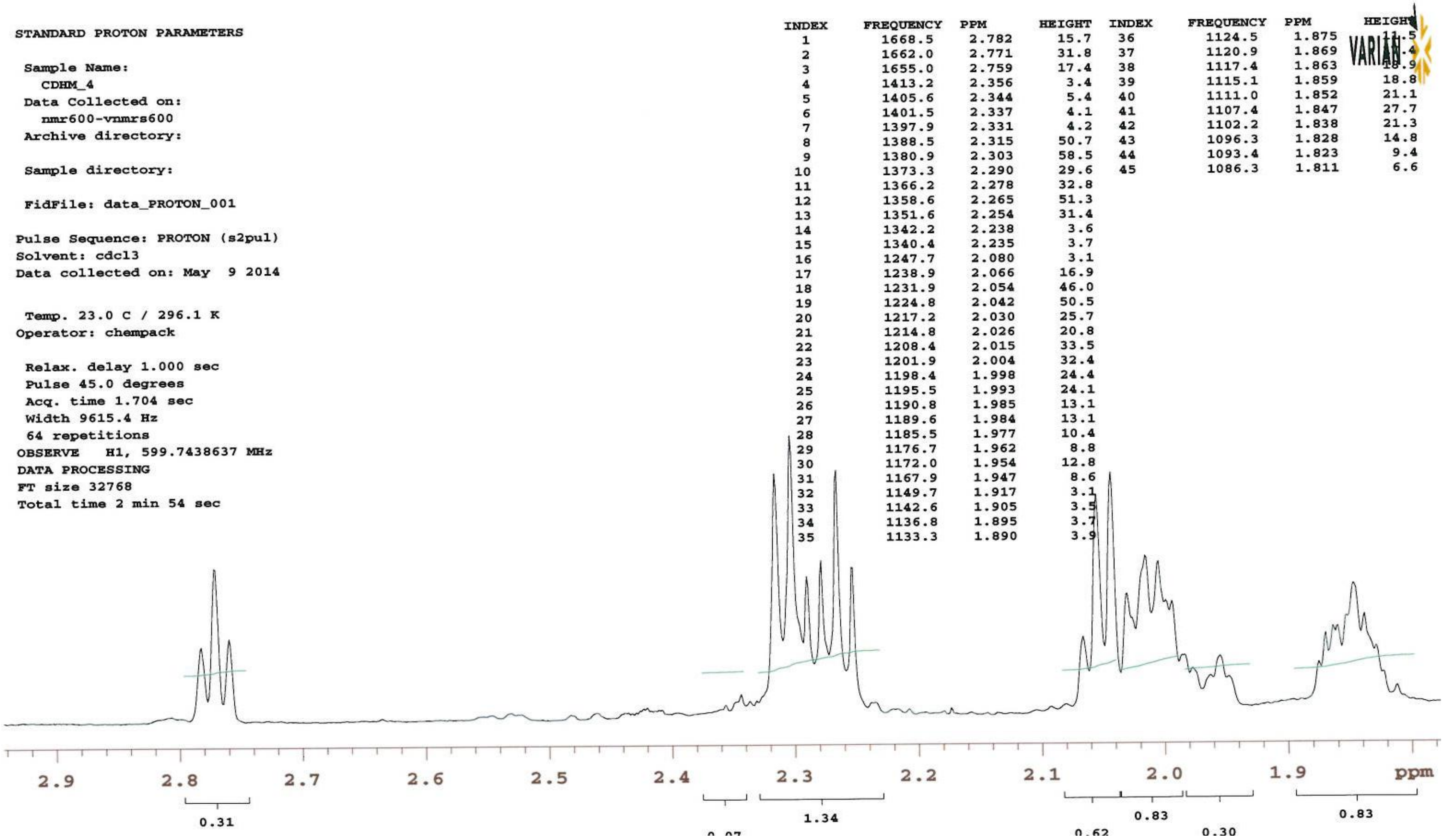

Fig. 23. ^{1}H-NMR spectrum of comp. 7 ($CDCl_3$, 600 MHz, 1.9-2.9)

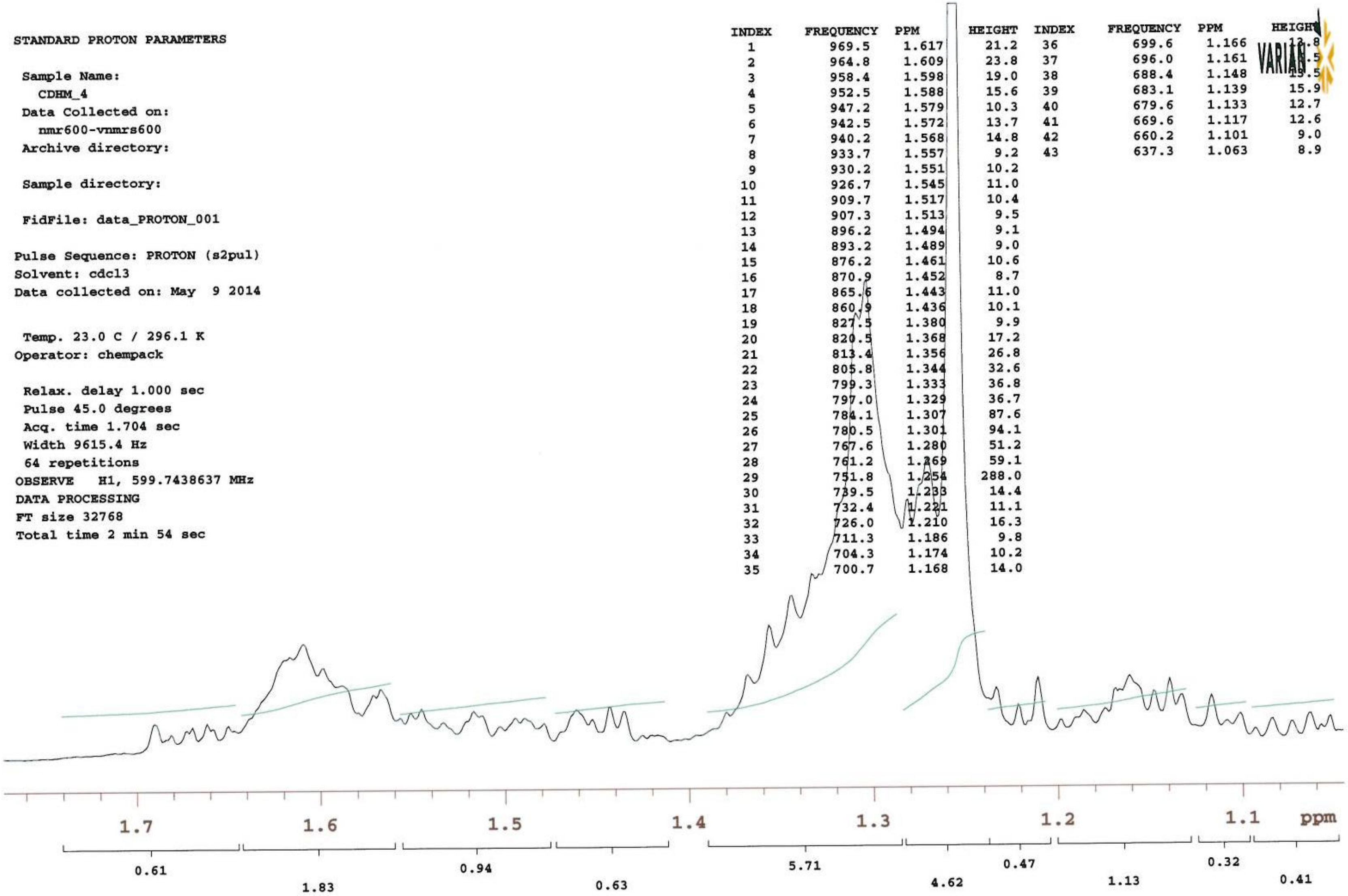

INDEX	FREQUENCY	PPM	HEIGHT	INDEX	FREQUENCY	PPM	HEIGHT
1	969.5	1.617	21.2	36	699.6	1.166	[illegible]
2	964.8	1.609	23.8	37	696.0	1.161	[illegible]
3	958.4	1.598	19.0	38	688.4	1.148	[illegible]
4	952.5	1.588	15.6	39	683.1	1.139	15.9
5	947.2	1.579	10.3	40	679.6	1.133	12.7
6	942.5	1.572	13.7	41	669.6	1.117	12.6
7	940.2	1.568	14.8	42	660.2	1.101	9.0
8	933.7	1.557	9.2	43	637.3	1.063	8.9
9	930.2	1.551	10.2				
10	926.7	1.545	11.0				
11	909.7	1.517	10.4				
12	907.3	1.513	9.5				
13	896.2	1.494	9.1				
14	893.2	1.489	9.0				
15	876.2	1.461	10.6				
16	870.9	1.452	8.7				
17	865.6	1.443	11.0				
18	860.9	1.436	10.1				
19	827.5	1.380	9.9				
20	820.5	1.368	17.2				
21	813.4	1.356	26.8				
22	805.8	1.344	32.6				
23	799.3	1.333	36.8				
24	797.0	1.329	36.7				
25	784.1	1.307	87.6				
26	780.5	1.301	94.1				
27	767.6	1.280	51.2				
28	761.2	1.269	59.1				
29	751.8	1.254	288.0				
30	739.5	1.233	14.4				
31	732.4	1.221	11.1				
32	726.0	1.210	16.3				
33	711.3	1.186	9.8				
34	704.3	1.174	10.2				
35	700.7	1.168	14.0				

Fig. 24. ^{1}H-NMR spectrum of comp. 7 ($CDCl_3$, 600 MHz, 1.1-1.7 ppm)

STANDARD PROTON PARAMETERS

Sample Name:
CDHM_4
Data Collected on:
nmr600-vnmrs600
Archive directory:

Sample directory:

FidFile: data_PROTON_001

Pulse Sequence: PROTON (s2pul)
Solvent: cdcl3
Data collected on: May 9 2014

Temp. 23.0 C / 296.1 K
Operator: chempack

Relax. delay 1.000 sec
Pulse 45.0 degrees
Acq. time 1.704 sec
Width 9615.4 Hz
64 repetitions
OBSERVE H1, 599.7438637 MHz
DATA PROCESSING
FT size 32768
Total time 2 min 54 sec

INDEX	FREQUENCY	PPM	HEIGHT
1	611.5	1.020	102.0
2	606.2	1.011	[illegible]
3	603.3	1.006	11.1
4	572.8	0.955	9.2
5	568.1	0.947	10.3
6	560.5	0.935	15.4
7	555.8	0.927	44.8
8	549.3	0.916	46.7
9	542.9	0.905	16.2
10	541.1	0.902	26.8
11	534.1	0.890	61.7
12	529.9	0.884	24.8
13	528.2	0.881	51.5
14	522.9	0.872	21.4
15	521.1	0.869	27.9
16	514.7	0.858	38.3
17	507.6	0.846	64.9
18	504.7	0.842	60.5
19	500.0	0.834	30.8
20	497.7	0.830	51.4
21	491.8	0.820	52.0
22	484.8	0.808	61.2
23	477.7	0.797	12.2
24	473.0	0.789	10.3
25	468.3	0.781	11.0
26	466.6	0.778	15.9
27	461.9	0.770	10.5
28	460.1	0.767	9.0
29	417.9	0.697	9.6
30	406.7	0.678	85.8

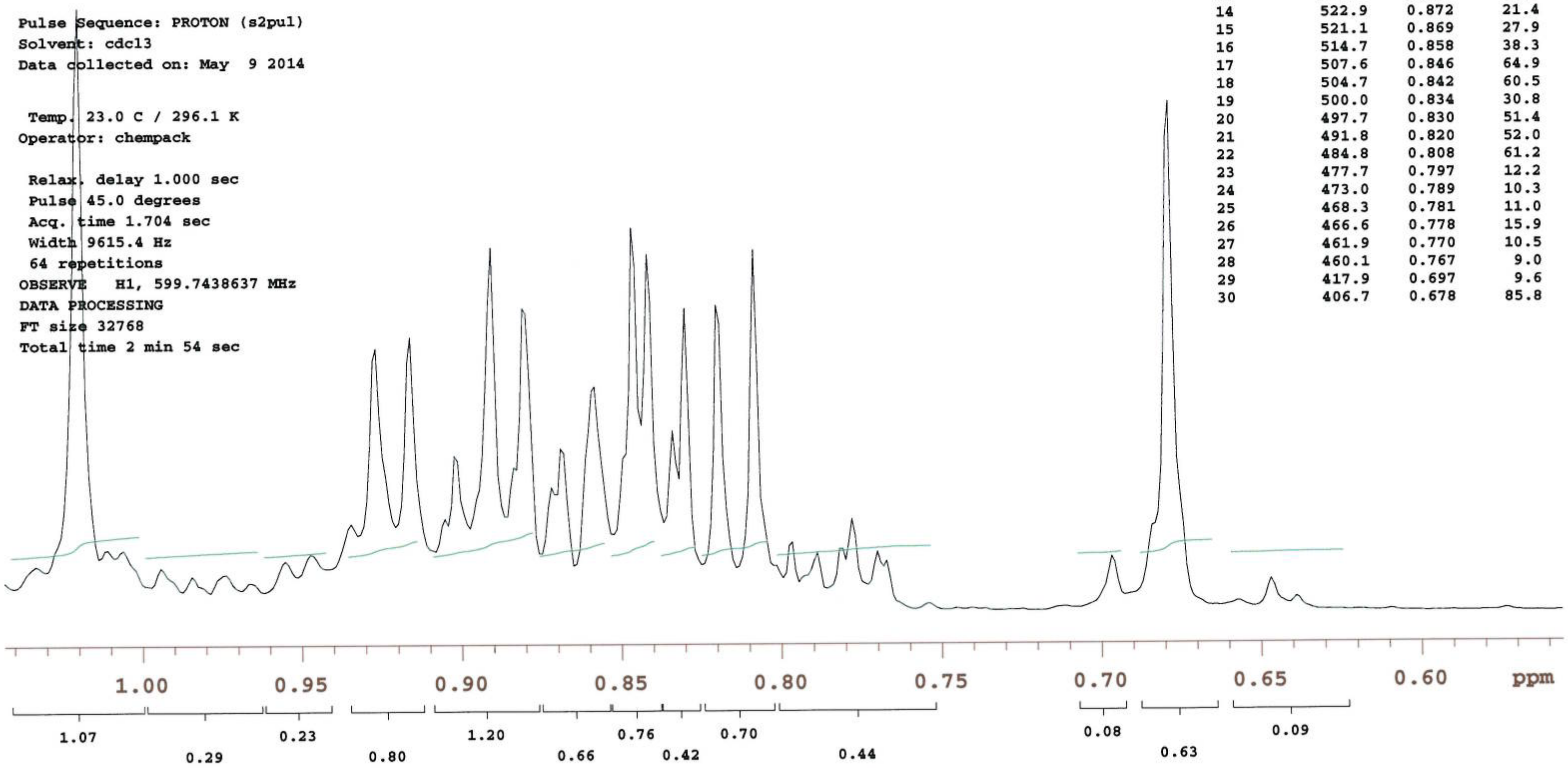

Fig. 25. ^{1}H-NMR spectrum of comp. 7 ($CDCl_3$, 600 MHz, 0.6-1.0 ppm)

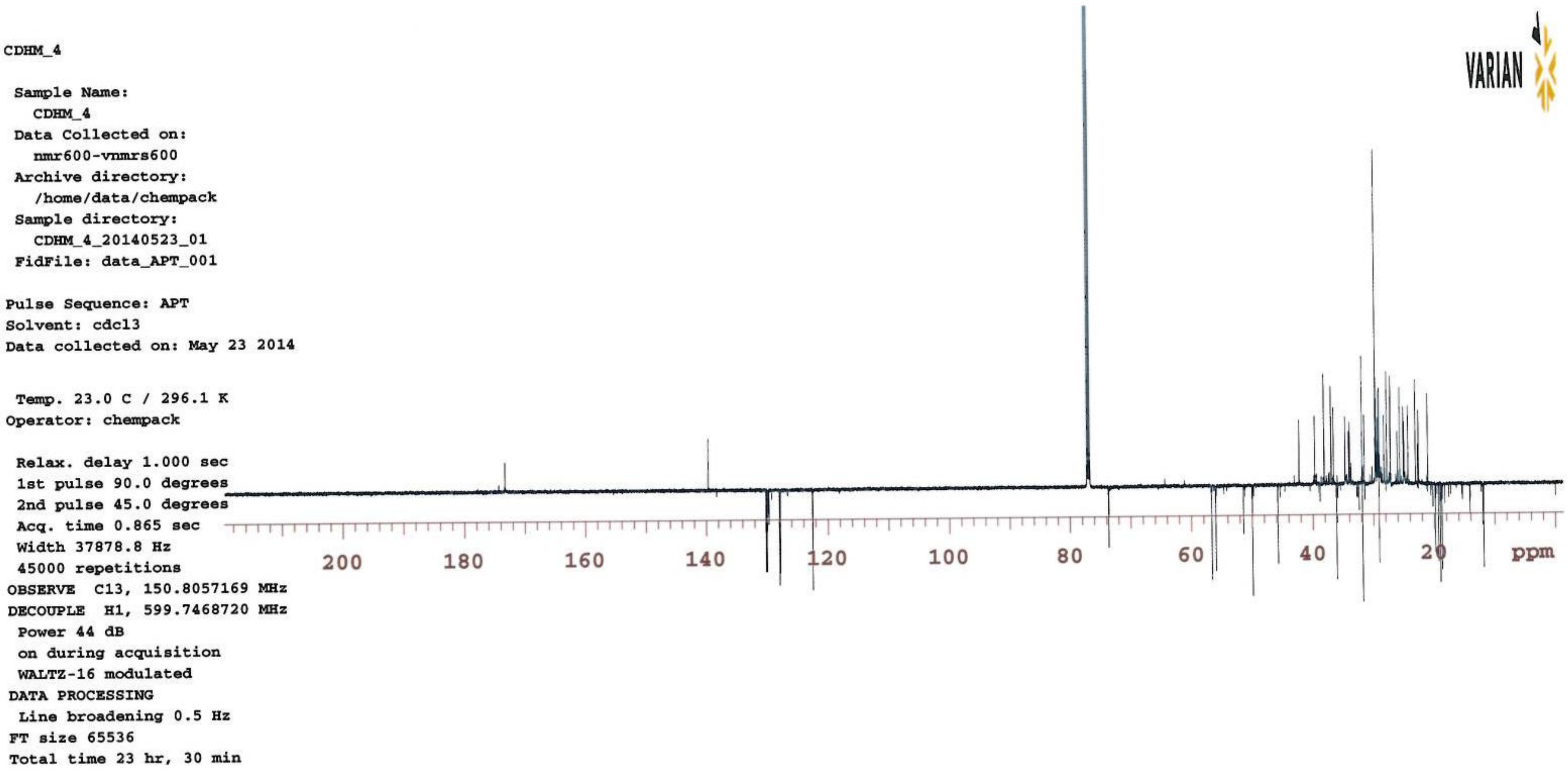

Fig. 26. ^{13}C-NMR spectrum of comp. 7 (CDCI$_3$, 125 MHz)

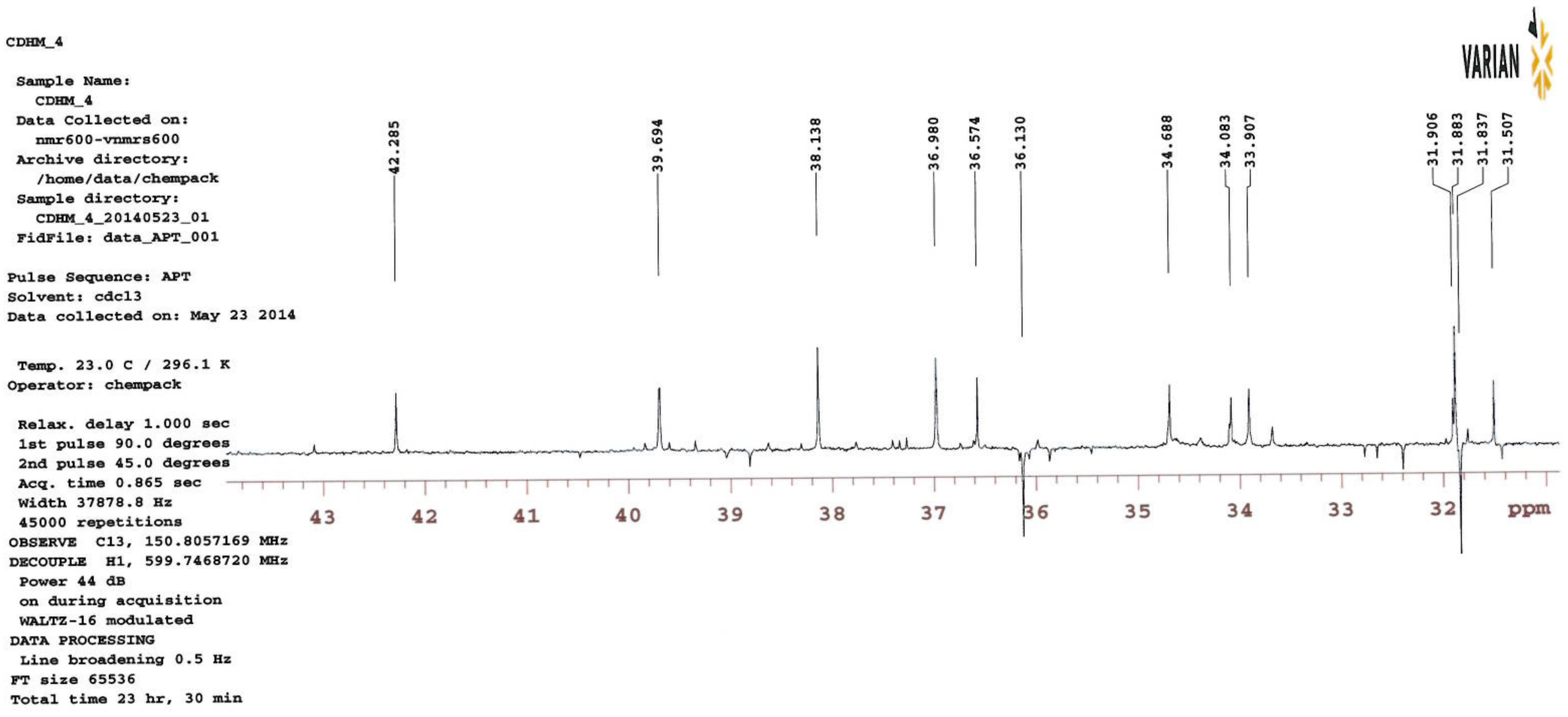

Fig. 27. ^{13}C-NMR spectrum of comp. 7 ($CDCl_3$, 125 MHz, 32-43 ppm)

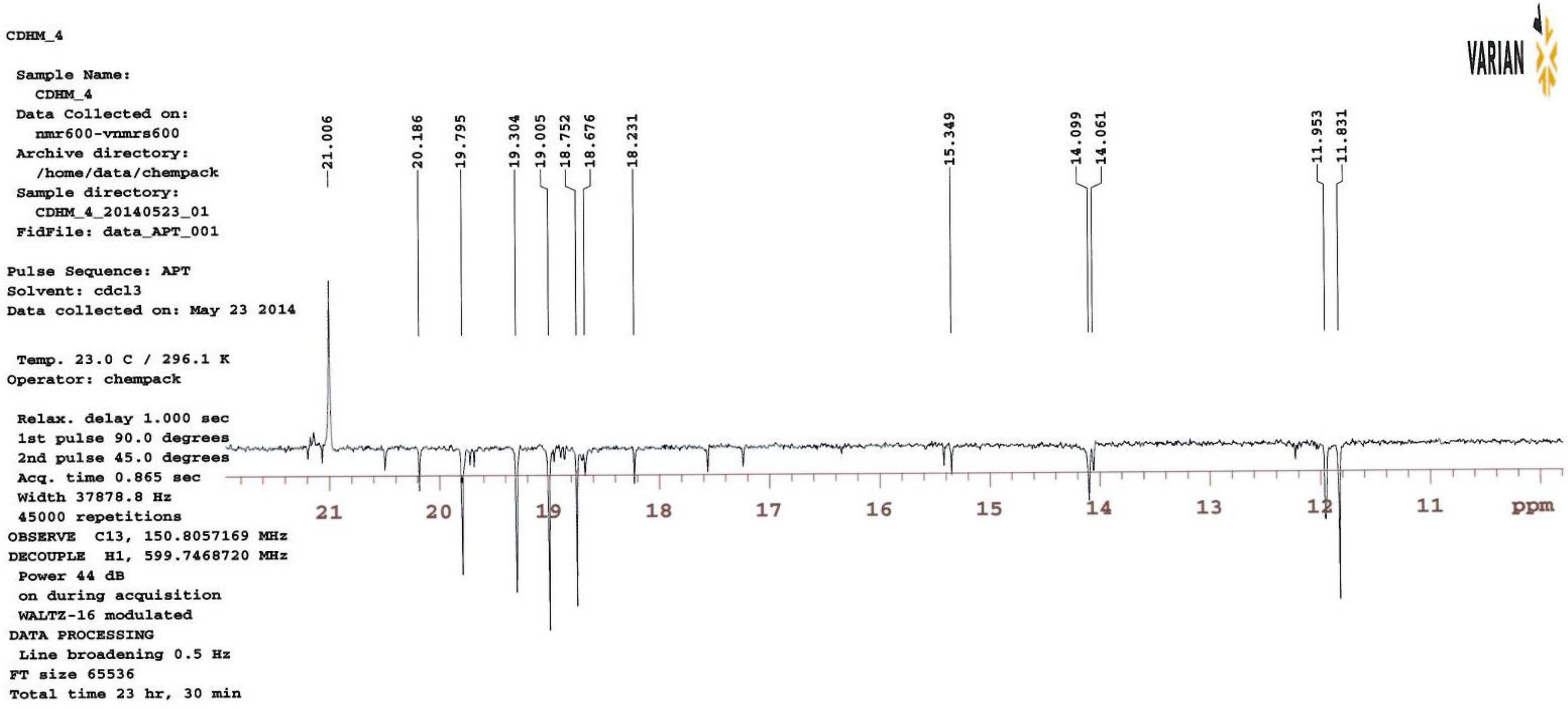

Fig. 28. ^{13}C-NMR spectrum of comp. 7 ($CDCl_3$, 125 MHz, 11-21 ppm)

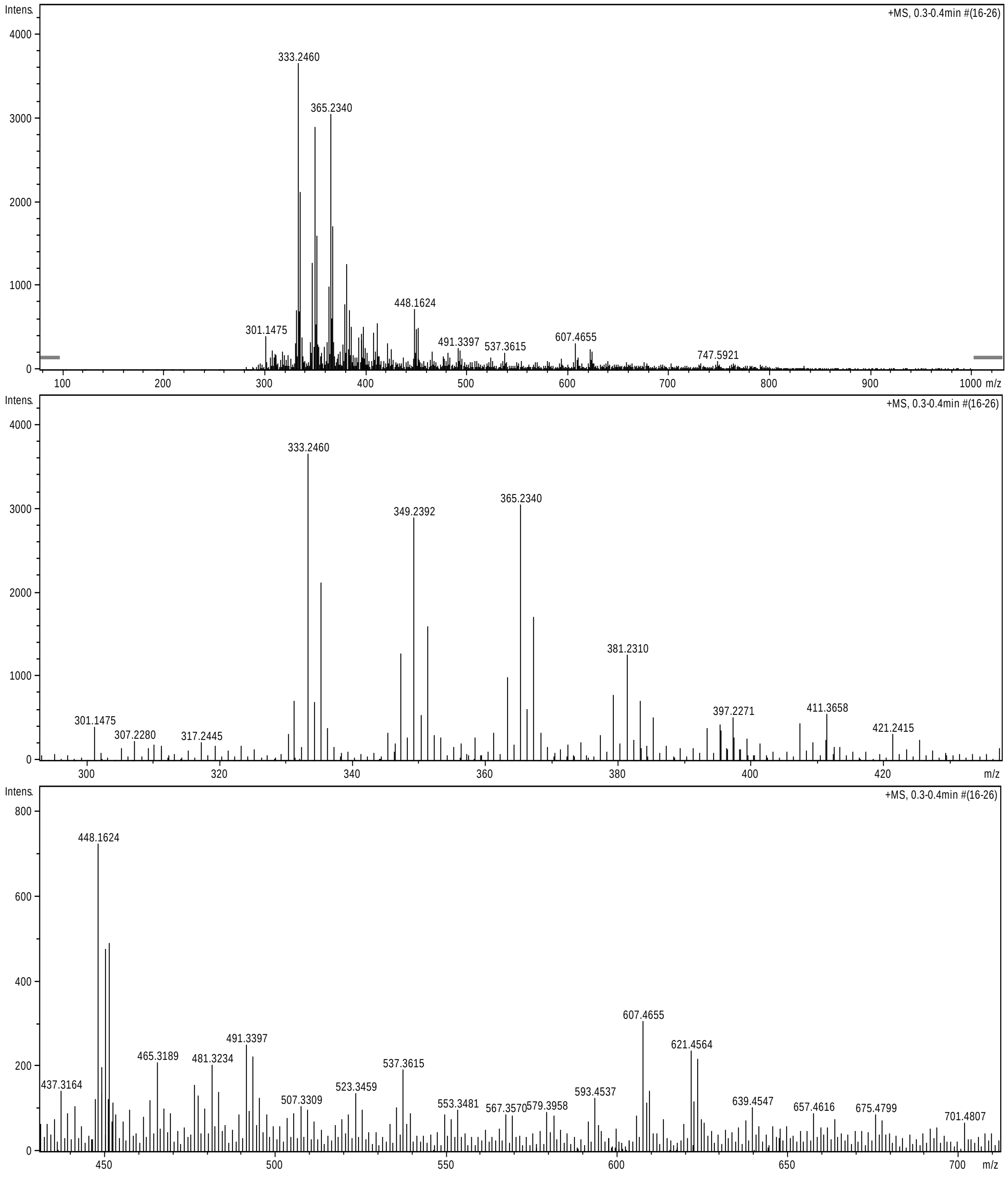

Fig. 29. MS spectrum of comp. 7

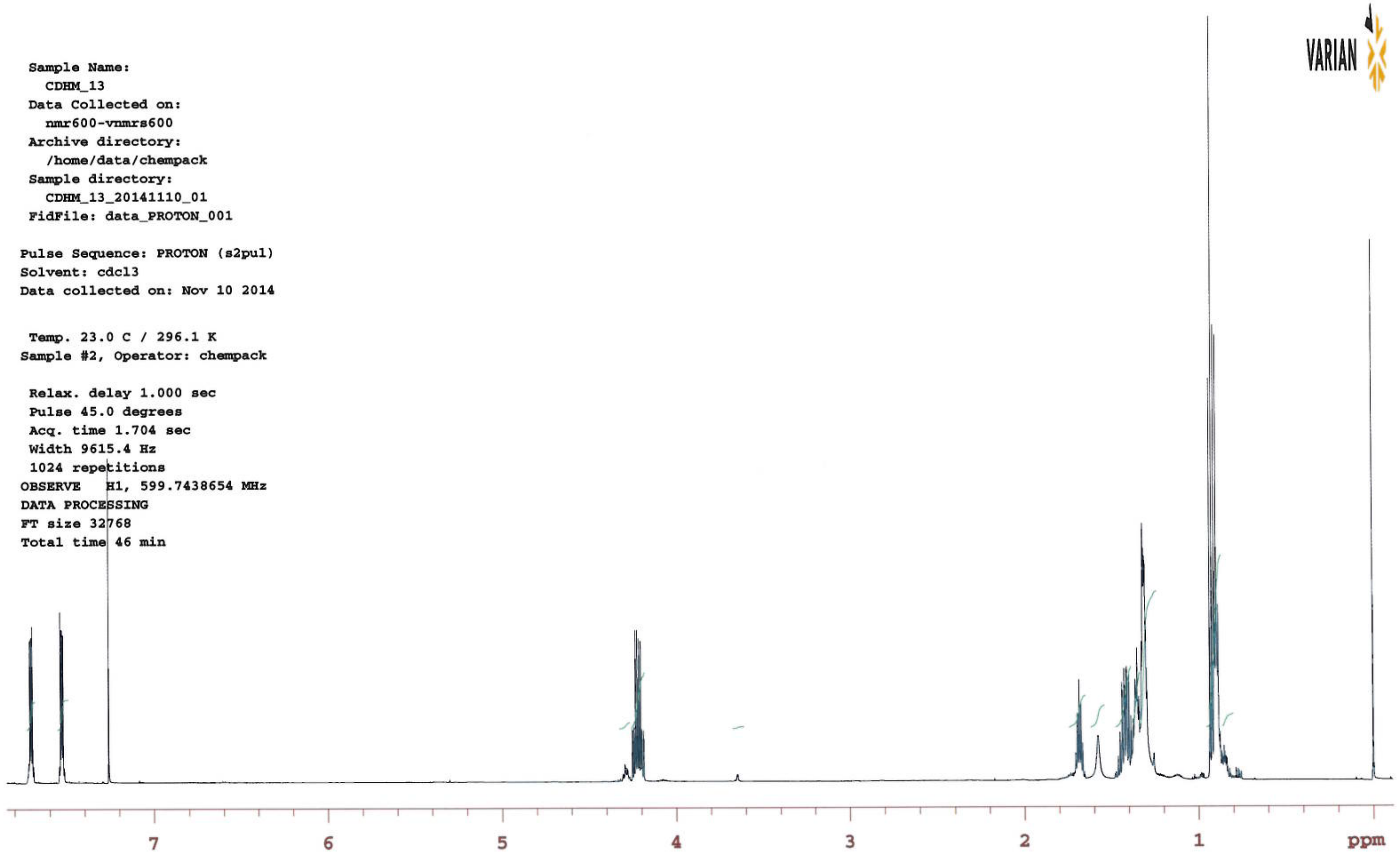

Fig. 30. ^{1}H-NMR spectrum of comp. 8 ($CDCl_3$, 600 MHz)

Sample Name:
CDHM_13
Data Collected on:
nmr600-vnmrs600
Archive directory:
/home/data/chempack
Sample directory:
CDHM_13_20141110_01
FidFile: data_PROTON_001

Pulse Sequence: PROTON (s2pul)
Solvent: cdcl3
Data collected on: Nov 10 2014

Temp. 23.0 C / 296.1 K
Sample #2, Operator: chempack

Relax. delay 1.000 sec
Pulse 45.0 degrees
Acq. time 1.704 sec
Width 9615.4 Hz
1024 repetitions
OBSERVE H1, 599.7438654 MHz
DATA PROCESSING
FT size 32768
Total time 46 min

INDEX	FREQUENCY	PPM	HEIGHT
1	4630.5	7.721	[illegible]
2	4626.9	7.715	[illegible]
3	4623.4	7.709	27.0
4	4621.1	7.705	27.3
5	4617.5	7.699	29.4
6	4614.0	7.693	3.0
7	4524.8	7.545	3.0
8	4521.3	7.539	32.1
9	4517.8	7.533	28.8
10	4515.4	7.529	28.7
11	4511.9	7.523	27.8
12	4355.2	7.262	61.1

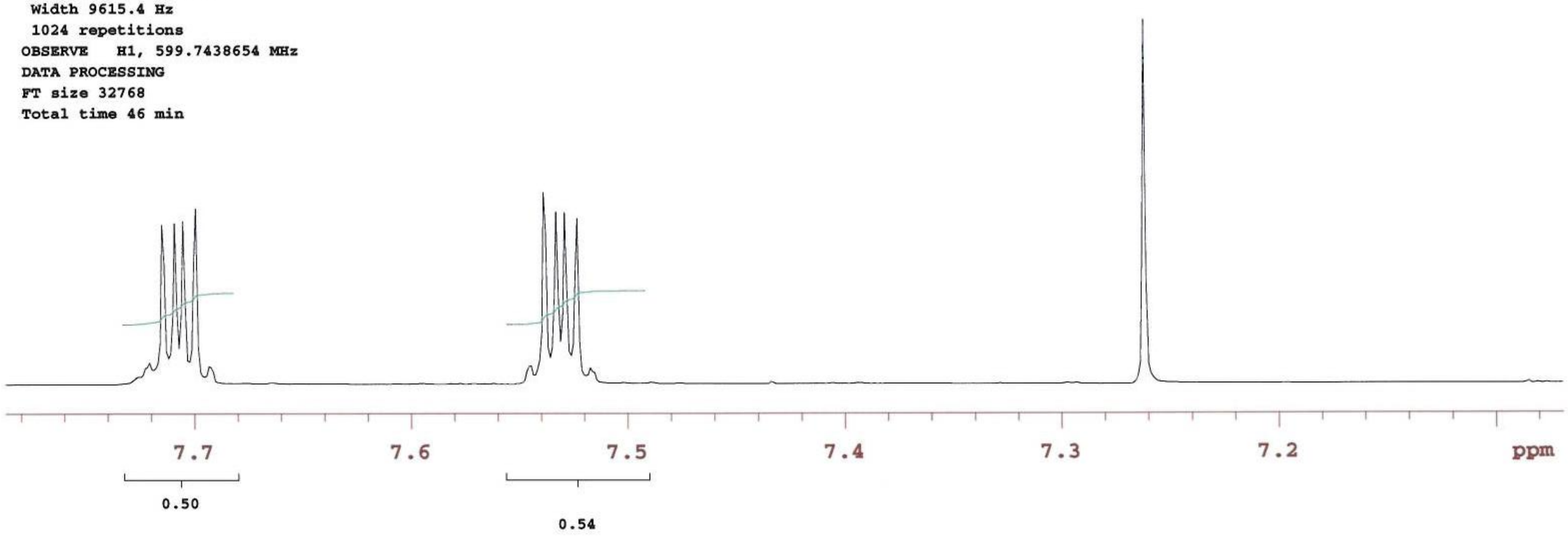

Fig. 31. ^{1}H-NMR spectrum of comp. 8 ($CDCl_3$, 600 MHz, 7.0-7.8 ppm)

Sample Name:
CDHM_13
Data Collected on:
nmr600-vnmrs600
Archive directory:
/home/data/chempack
Sample directory:
CDHM_13_20141110_01
FidFile: data_PROTON_001

Pulse Sequence: PROTON (s2pul)
Solvent: cdcl3
Data collected on: Nov 10 2014

Temp. 23.0 C / 296.1 K
Sample #2, Operator: chempack

Relax. delay 1.000 sec
Pulse 45.0 degrees
Acq. time 1.704 sec
Width 9615.4 Hz
1024 repetitions
OBSERVE H1, 599.7438654 MHz
DATA PROCESSING
FT size 32768
Total time 46 min

INDEX	FREQUENCY	PPM	HEIGHT
1	2575.8	4.295	[illegible]
2	2550.0	4.252	[illegible]
3	2544.1	4.242	10.2
4	2538.8	4.233	28.4
5	2533.0	4.223	28.5
6	2527.7	4.215	26.9
7	2521.8	4.205	26.5
8	2516.5	4.196	9.8
9	2510.7	4.186	9.6

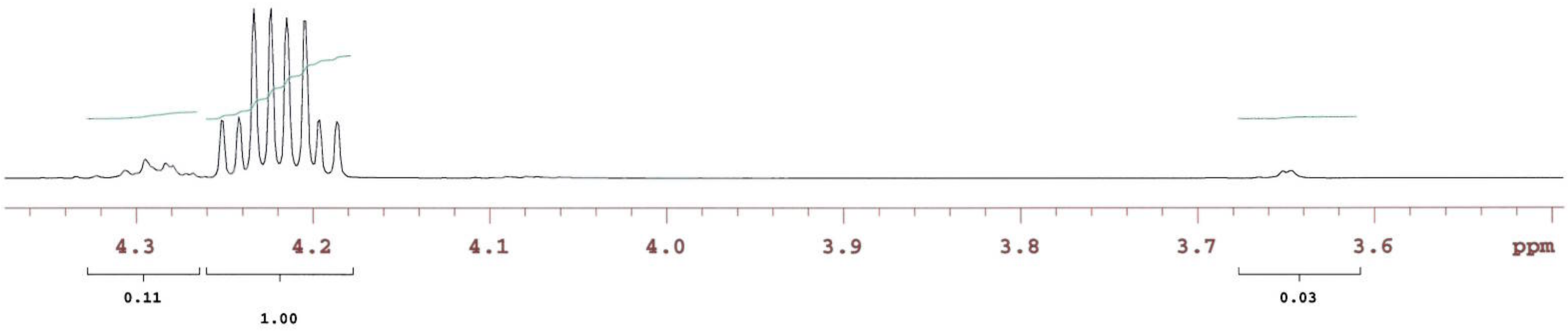

Fig. 32. ^{1}H-NMR spectrum of comp. 8 ($CDCl_3$, 600 MHz, 3.6-4.3 ppm)

Sample Name:
CDHM_13
Data Collected on:
nmr600-vnmrs600
Archive directory:
/home/data/chempack
Sample directory:
CDHM_13_20141110_01
FidFile: data_PROTON_001

Pulse Sequence: PROTON (s2pul)
Solvent: cdcl3
Data collected on: Nov 10 2014

Temp. 23.0 C / 296.1 K
Sample #2, Operator: chempack

Relax. delay 1.000 sec
Pulse 45.0 degrees
Acq. time 1.704 sec
Width 9615.4 Hz
1024 repetitions
OBSERVE H1, 599.7438654 MHz
DATA PROCESSING
FT size 32768
Total time 46 min

INDEX	FREQUENCY	PPM	HEIGHT
1	1022.9	1.706	5.1
2	1016.5	1.695	12.7
3	1010.6	1.685	19.0
4	1004.1	1.674	14.6
5	998.3	1.665	7.0
6	945.5	1.576	8.4
7	875.6	1.460	4.5
8	869.2	1.449	9.1
9	862.1	1.437	18.4
10	859.8	1.434	6.7
11	854.5	1.425	21.1
12	852.1	1.421	17.8
13	848.0	1.414	21.1
14	845.7	1.410	21.4
15	840.4	1.401	8.3
16	838.1	1.397	20.0
17	831.0	1.386	12.1
18	828.1	1.381	5.4
19	823.4	1.373	11.6
20	819.3	1.366	12.2
21	816.3	1.361	19.0
22	814.0	1.357	16.6
23	809.9	1.350	24.9
24	808.1	1.347	15.2
25	806.4	1.345	12.5
26	803.4	1.340	15.8
27	799.3	1.333	10.6
28	794.0	1.324	26.1
29	789.9	1.317	48.4
30	787.0	1.312	43.7
31	783.5	1.306	42.2
32	778.8	1.299	30.5
33	775.9	1.294	21.1
34	770.6	1.285	6.8
35	765.9	1.277	4.4
36	760.6	1.268	[illegible]
37	752.4	1.254	[illegible]
38	559.9	0.934	[illegible]
39	552.8	0.922	144.0
40	545.2	0.909	85.8
41	541.1	0.902	22.8
42	537.6	0.896	83.9
43	530.5	0.885	33.1
44	527.0	0.879	8.6
45	524.7	0.875	9.2
46	521.1	0.869	3.2
47	518.8	0.865	5.5
48	515.9	0.860	3.5
49	511.2	0.852	6.5
50	508.2	0.847	5.4
51	504.1	0.841	4.4
52	501.8	0.837	4.1

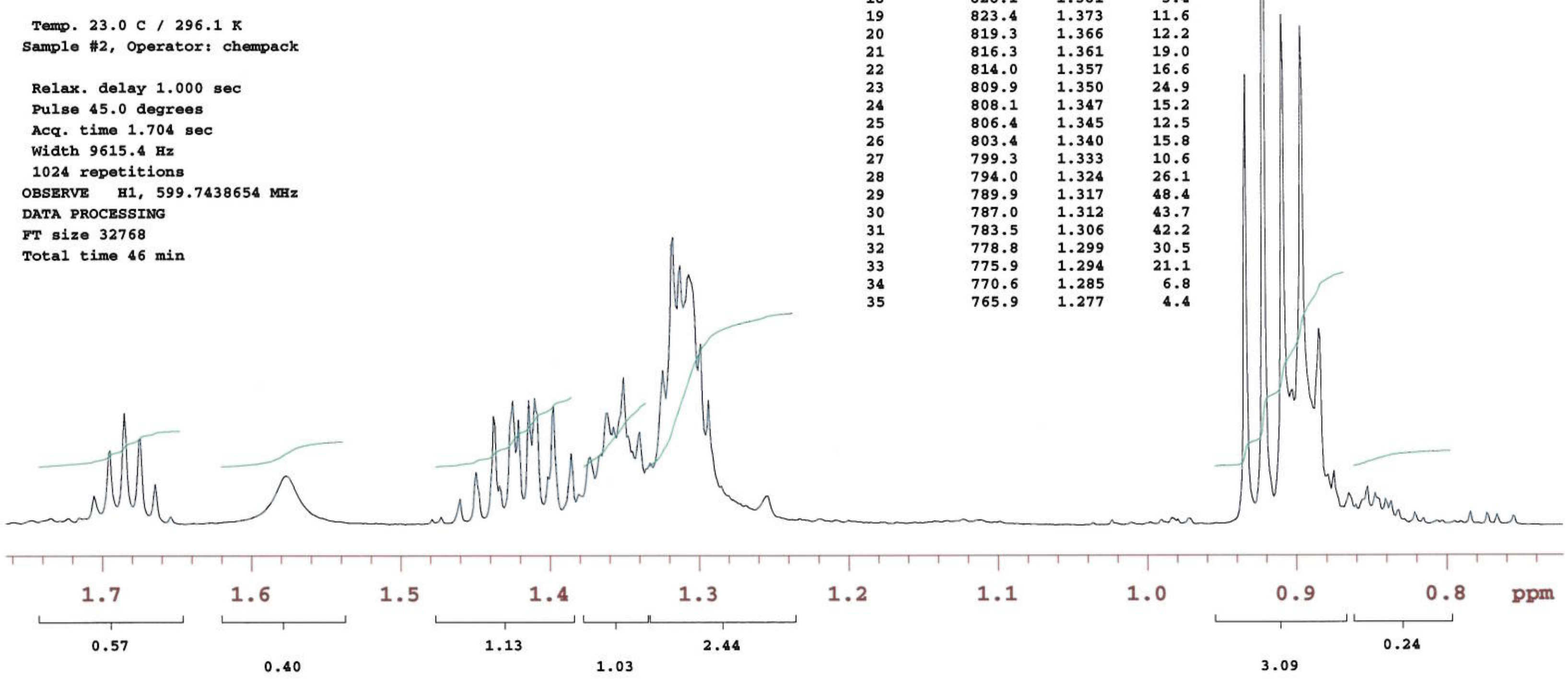

Fig. 33. ^{1}H-NMR spectrum of comp. 8 (CDCI$_3$, 600 MHz, 0.8-1.7 ppm)

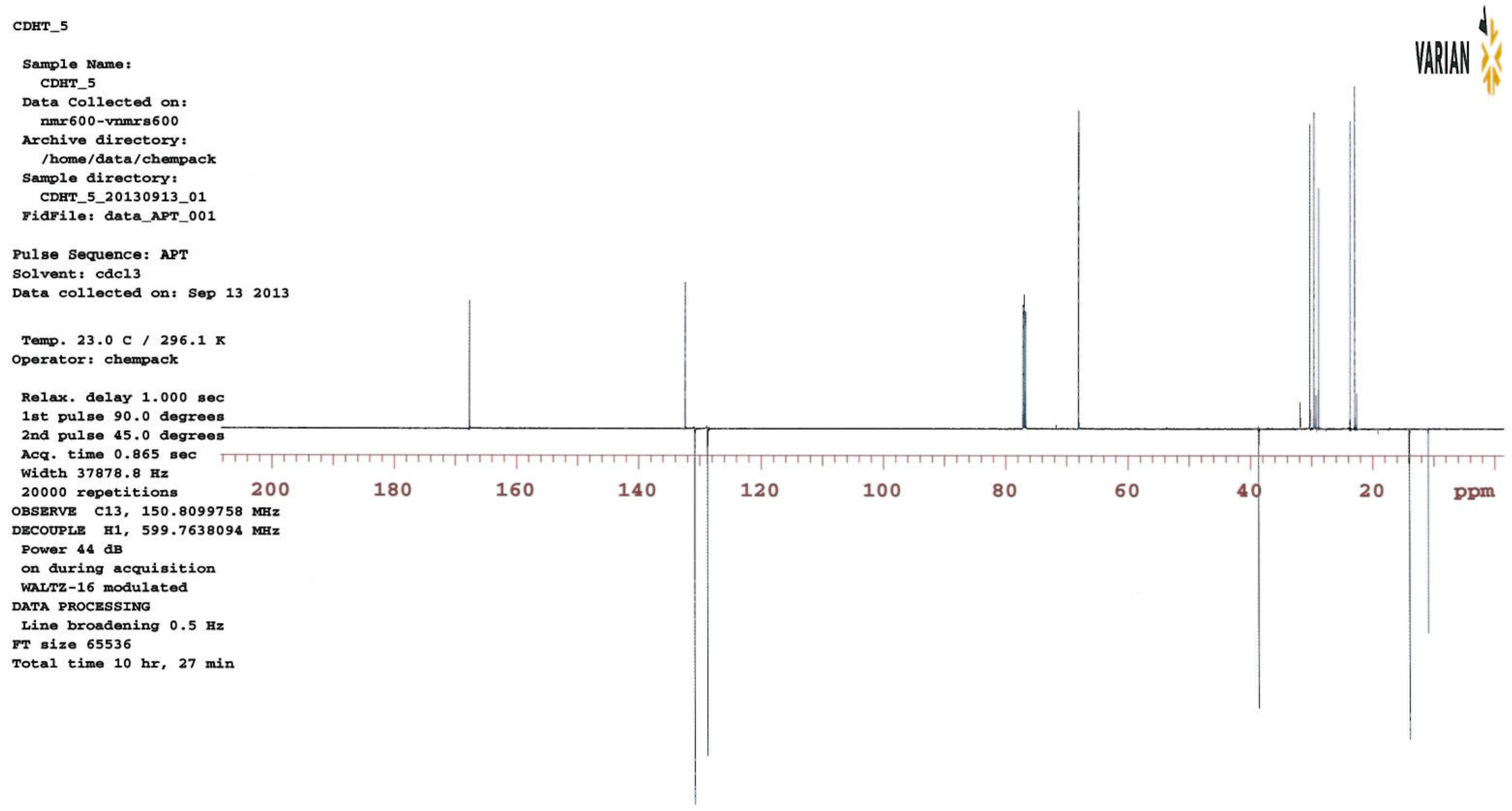

Fig. 34. ^{13}C-NMR spectrum of comp. 8 ($CDCl_3$, 125 MHz)

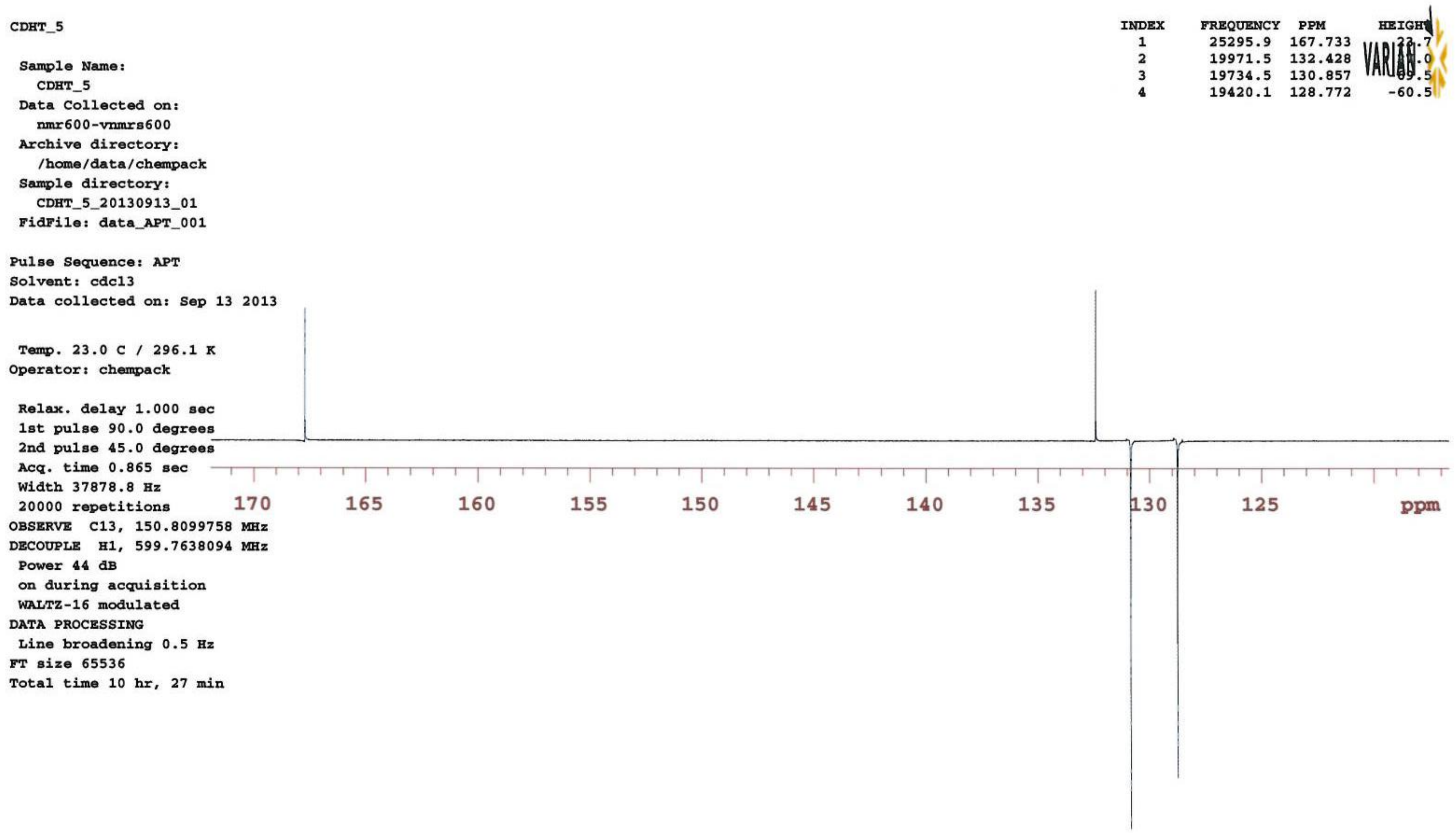

Fig. 35. ^{13}**C-NMR spectrum of comp. 8 (CDCl$_3$, 125 MHz, 125-170 ppm)**

CDHT_5

Sample Name:
CDHT_5
Data Collected on:
nmr600-vnmrs600
Archive directory:
/home/data/chempack
Sample directory:
CDHT_5_20130913_01
FidFile: data_APT_001

Pulse Sequence: APT
Solvent: cdcl3
Data collected on: Sep 13 2013

Temp. 23.0 C / 296.1 K
Operator: chempack

Relax. delay 1.000 sec
1st pulse 90.0 degrees
2nd pulse 45.0 degrees
Acq. time 0.865 sec
Width 37878.8 Hz
20000 repetitions
OBSERVE C13, 150.8099758 MHz
DECOUPLE H1, 599.7638094 MHz
Power 44 dB
on during acquisition
WALTZ-16 modulated
DATA PROCESSING
Line broadening 0.5 Hz
FT size 65536
Total time 10 hr, 27 min

INDEX	FREQUENCY	PPM	HEIGHT
1	10272.9	68.118	[illegible]
2	5836.3	38.700	[illegible]
3	4812.1	31.908	[illegible]

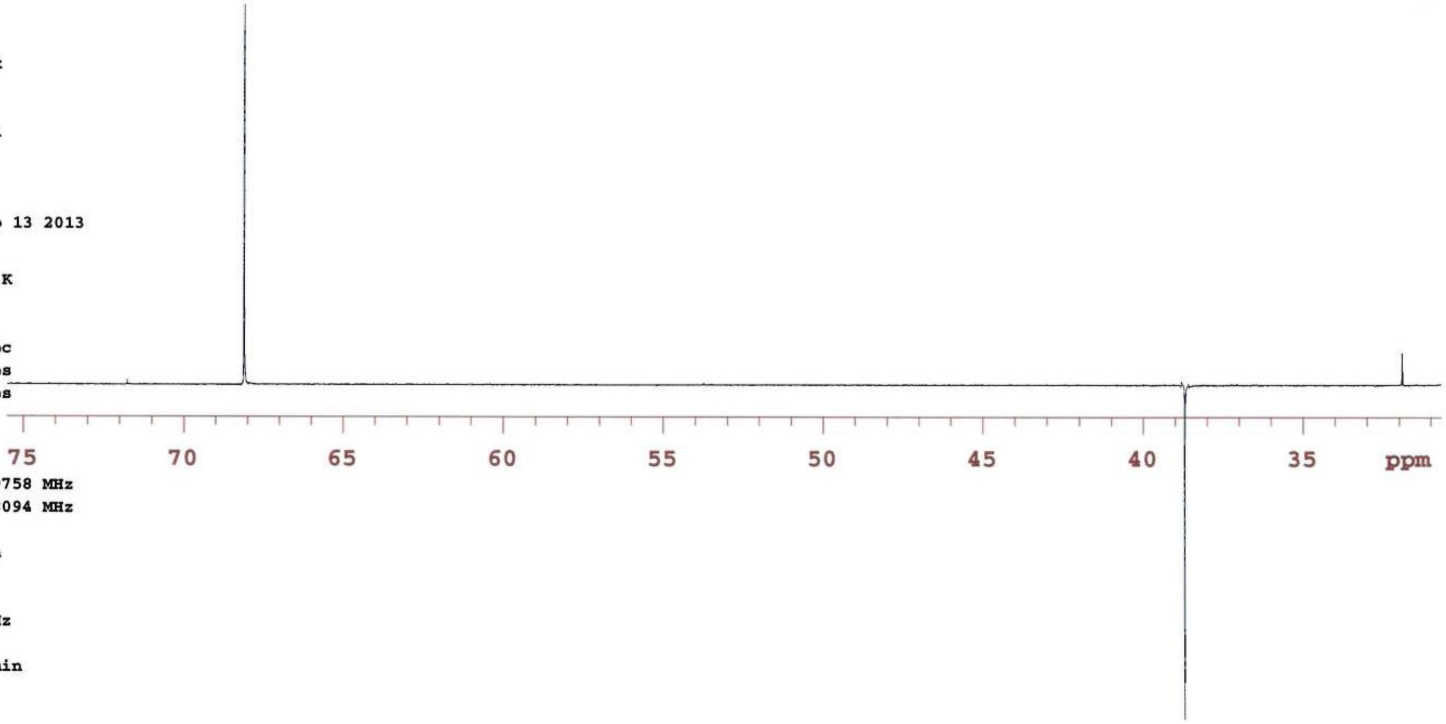

Fig. 36. ^{13}C-NMR spectrum of comp. 8 ($CDCl_3$, 125 MHz, 35-75 ppm)

CDHT_5

Sample Name:
CDHT_5
Data Collected on:
nmr600-vnmrs600
Archive directory:
/home/data/chempack
Sample directory:
CDHT_5_20130913_01
FidFile: data_APT_001

Pulse Sequence: APT
Solvent: cdcl3
Data collected on: Sep 13 2013

Temp. 23.0 C / 296.1 K
Operator: chempack

Relax. delay 1.000 sec
1st pulse 90.0 degrees
2nd pulse 45.0 degrees
Acq. time 0.865 sec
Width 37878.8 Hz
20000 repetitions
OBSERVE C13, 150.8099758 MHz
DECOUPLE H1, 599.7638094 MHz
Power 44 dB
on during acquisition
WALTZ-16 modulated
DATA PROCESSING
Line broadening 0.5 Hz
FT size 65536
Total time 10 hr, 27 min

INDEX	FREQUENCY	PPM	HEIGHT
1	4575.1	30.337	[illegible]
2	4475.7	29.678	[illegible]
3	4469.9	29.640	[illegible]
4	4424.9	29.341	6.2
5	4359.0	28.904	44.4
6	3577.5	23.722	56.8
7	3463.1	22.963	63.2
8	3419.2	22.672	6.5
9	2125.6	14.095	-6.4
10	2115.2	14.026	-57.3
11	1649.4	10.937	-37.7

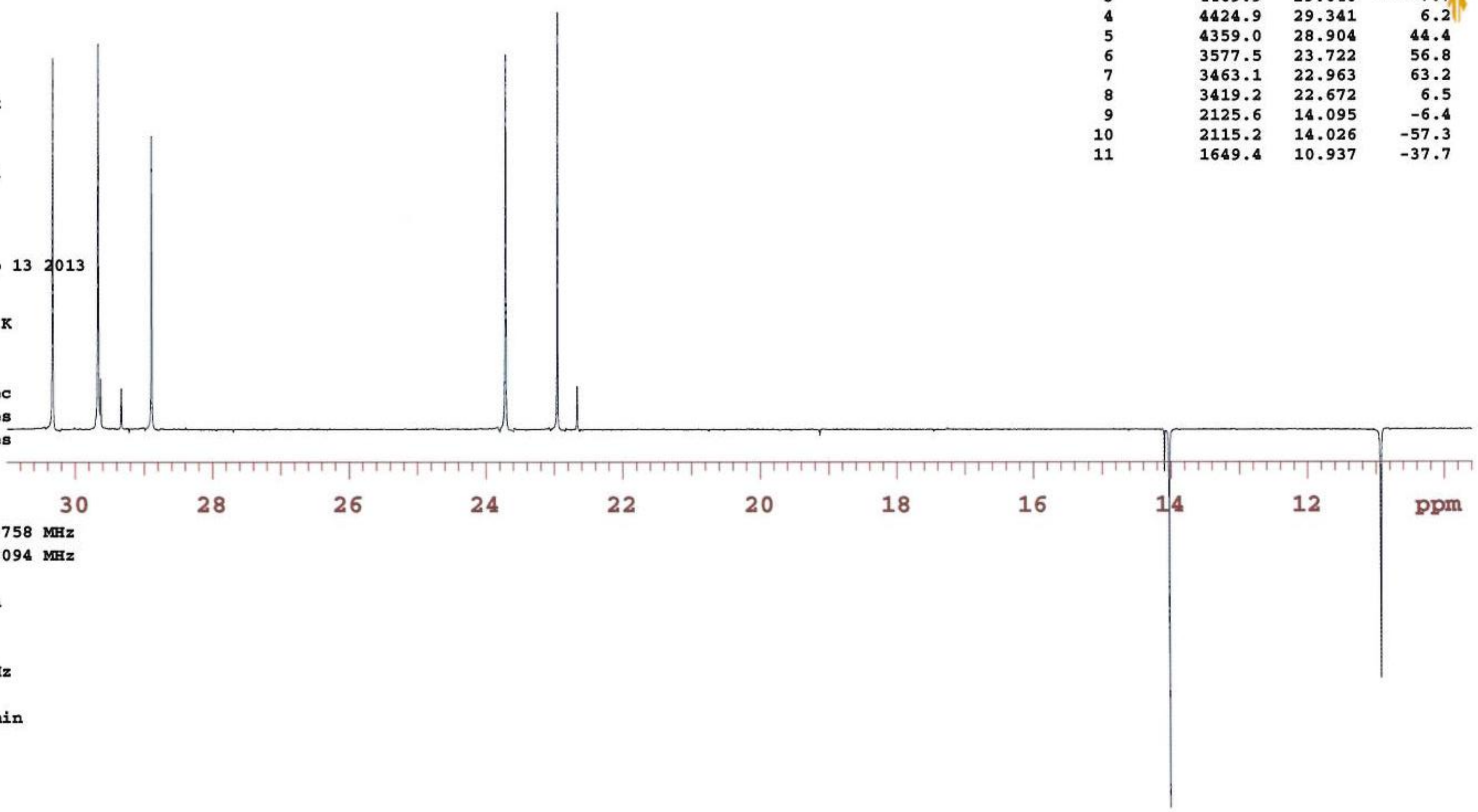

Fig. 36. ^{13}C-NMR spectrum of comp. 8 ($CDCl_3$, 125 MHz, 10-30 ppm)

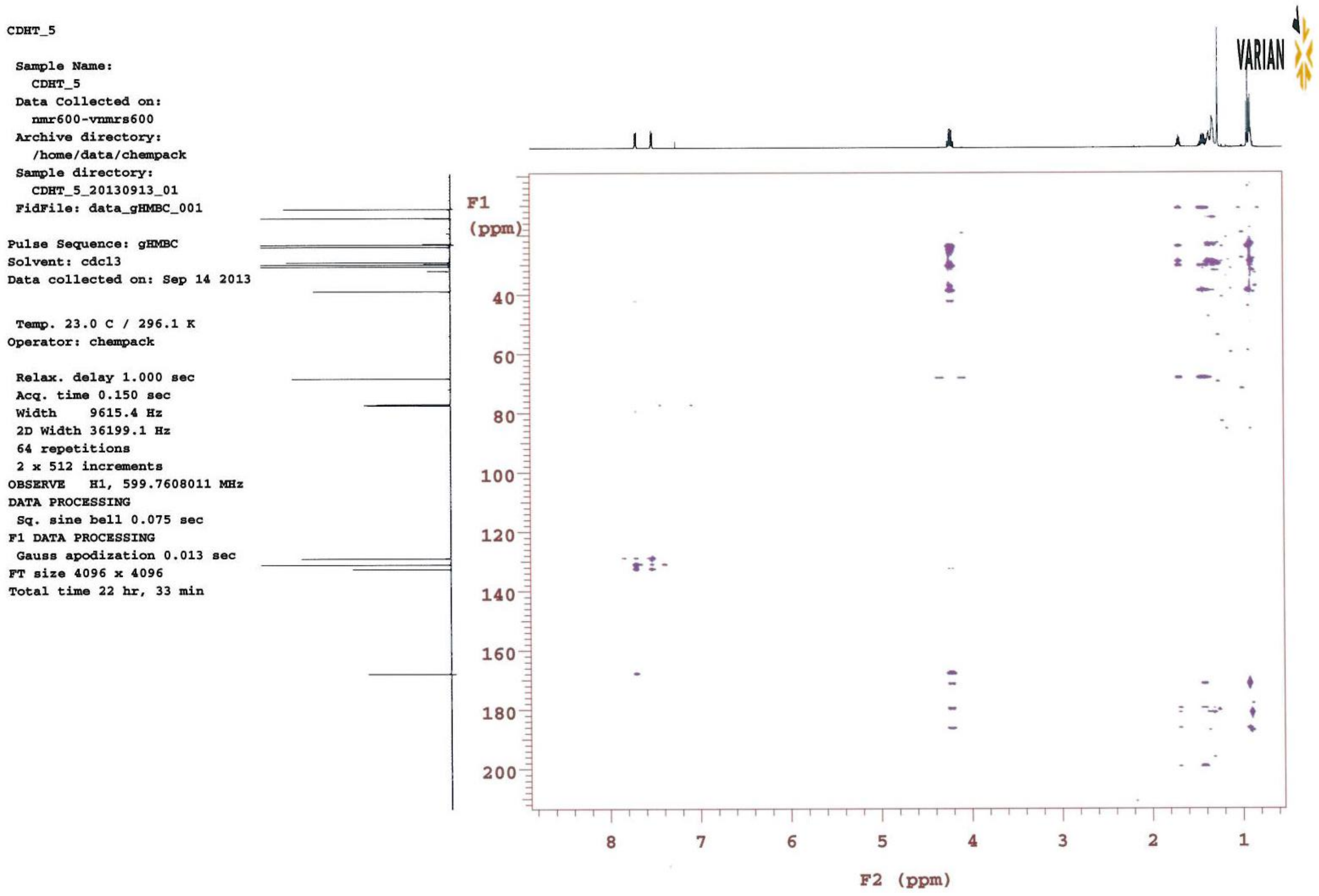

Fig. 37. HMBC spectrum of comp. 8 (CDCI$_3$, 125 MHz)

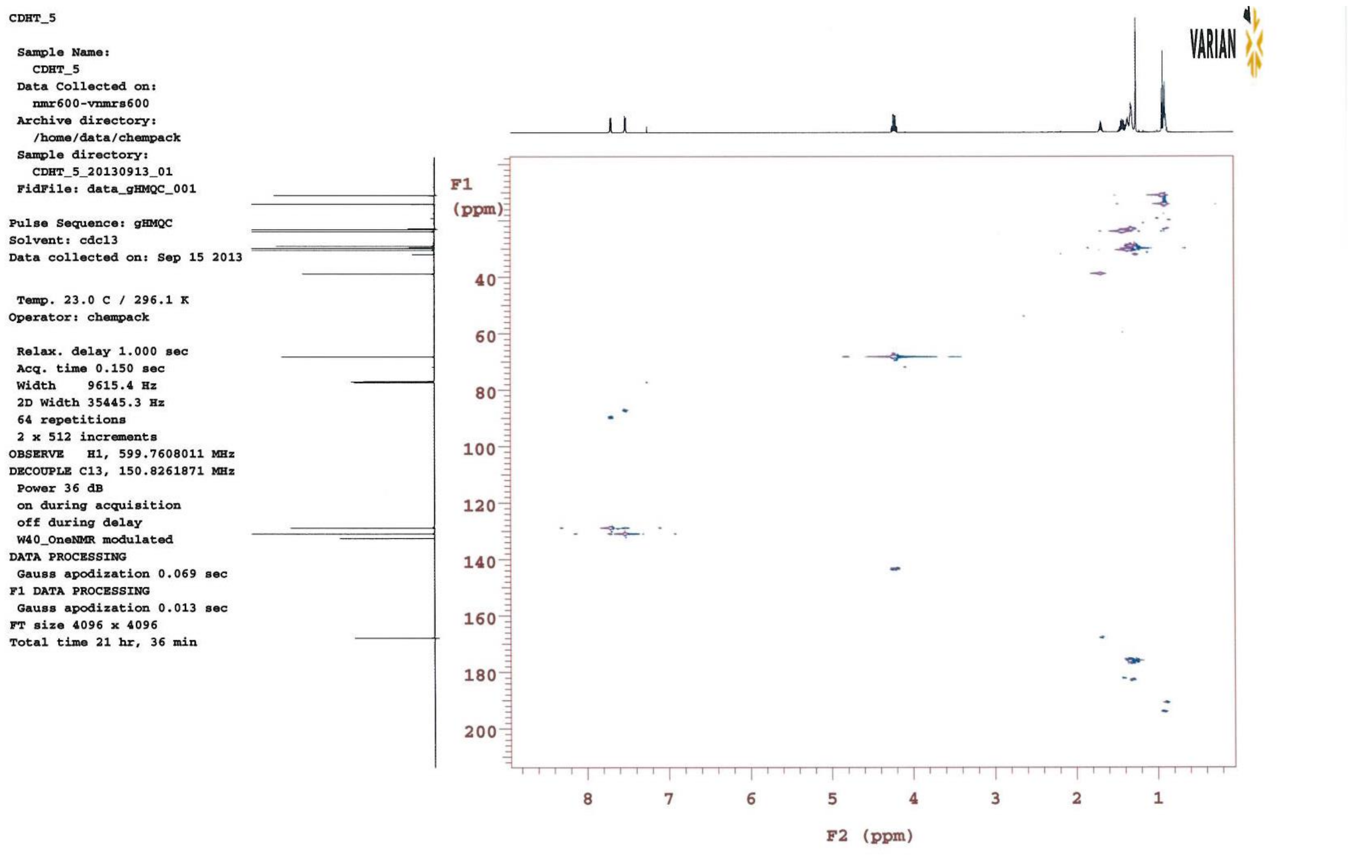

Fig. 38. HMQC spectrum of comp. 8 ($CDCl_3$, 125 MHz)

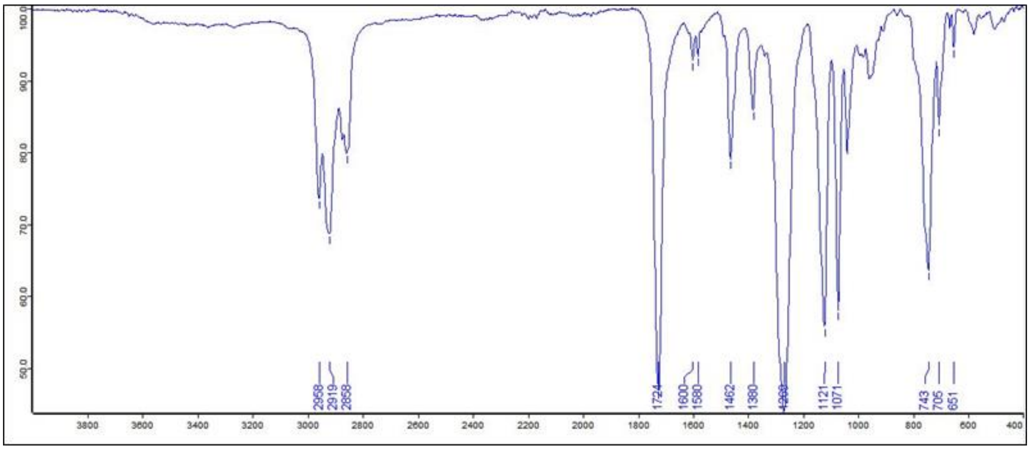

Fig. 39. IR spectrum of comp. 8

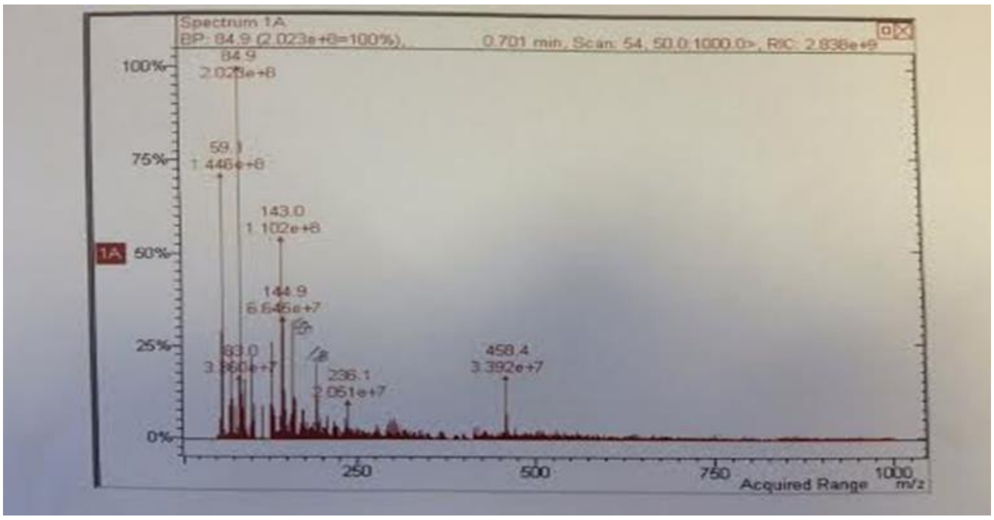

Fig. 40. MS spectrum of comp. 8

Table S1. Primer sequences of the selected human genes

GeneBank	Gene	F_Sequence (5'->3')	R_Sequence(5'->3')	Tm
NM_002985	CCL5	CAGTCGTCTTTGTCACCCGA	AGAGCAAGCAGAAACAGGCA	62^{0}C
NM_001565	CXCL10	ACCAGAGGGGAGCAAAATCG	GGAAGTGATGGGAGAGGCAG	62^{0}C
NM_002416	CXCL9	GGCTCTTTCCTGGCTACTCC	TCCCTGGTCCCTGTAGTGAG	61^{0}C
NM_001530	HIF1A	GGCGCGAACGACAAGAAAAA	GTGGCAACTGATGAGCAAGC	61^{0}C
NM_000600	IL6	ACTCACCTCTTCAGAACGAATTG	CCATCTTTGGAAGGTTCAGGTTG	59^{0}C
NM_003998	NFKB1	TCGCGCTGAGTATAAAAGCC	GGCAAAGTTTCGTGGATGCG	61^{0}C
NM_000594	TNF	TGGGATCATTGCCCTGTGAG	GGTGTCTGAAGGAGGGGGTA	62^{0}C
NM_001101	ACTB	GCCGCCAGCTCACCAT	GATGCCTCTCTTGCTCTGGG	59^{0}C
